# Supercritical Fluid‐Facilitated Exfoliation and Processing of 2D Materials

**DOI:** 10.1002/advs.201901084

**Published:** 2019-07-24

**Authors:** Zhenyu Sun, Qun Fan, Mingli Zhang, Shizhen Liu, Hengcong Tao, John Texter

**Affiliations:** ^1^ State Key Laboratory of Organic–Inorganic Composites Beijing University of Chemical Technology Beijing 100029 P. R. China; ^2^ School of Engineering Technology Eastern Michigan University Ypsilanti MI 48197 USA

**Keywords:** 2D materials, application, exfoliation, modification, supercritical fluids

## Abstract

Since the first intercalation of layered silicates by using supercritical CO_2_ as a processing medium, considerable efforts have been dedicated to intercalating and exfoliating layered two‐dimensional (2D) materials in various supercritical fluids (SCFs) to yield single‐ and few‐layer nanosheets. Here, recent work in this area is highlighted. Motivating factors for enhancing exfoliation efficiency and product quality in SCFs, mechanisms for exfoliation and dispersion in SCFs, as well as general metrics applied to assess quality and processability of exfoliated 2D materials are critically discussed. Further, advances in formation and application of 2D material–based composites with assistance from SCFs are presented. These discussions address chemical transformations accompanying SCF processing such as doping, covalent surface modification, and heterostructure formation. Promising features, challenges, and routes to expanding SCF processing techniques are described.

## 1 Introduction

Nanosheet materials are creating much excitement in advanced materials research because of their essentially 2D sheet structures and diversity of chemical characteristics. We briefly discuss their compositional diversity and approaches being taken for their syntheses and processing. Supercritical fluids (SCFs) are introduced, and their properties are reviewed. Various applications of SCFs to exfoliation are presented with emphasis on opportunities to process without chemically modifying 2D materials, as well as examples where a chemical change (such as redox chemistry, sheet scission) is a natural outcome of using particular SCFs.

Following this introduction, we focus on briefly articulating the predominant SCF‐based or ‐assisted processing methods with accompanying applications made to nanosheet materials. Many of these methods have already found application in various industries for microparticle and nanoparticle processing. Next, we examine exfoliation using SCFs and specific types of nanosheet processing methods that mostly have to do with graphene (G) and graphene oxide (GO) processing. We also introduce early simulations of SCF‐based exfoliation processing to present a primary example of sophisticated molecular potential barriers emanating from Israelachvili's solvation force work from the last century. Our most lengthy section addresses diverse applications of SCF‐based nanosheet processing that impact many industrially essential topics. We conclude with a summary and try to describe some challenges materials scientists and engineers face in expanding SCF processing of layered materials.

### 1.1 Nanosheets and Breadth of 2D Materials

2D materials are referred to as nanosheets with atomic thickness and significantly larger lateral sizes. Thinning of layered materials down to a single layer or few layers can lead to enhanced or unusual electronic, optical, and mechanical properties as well as large specific surface area and chemical reactivity due to electron confinement in two dimensions.

The past decade has witnessed a rapid development of 2D compounds in terms of variety and number of materials following the discovery of graphene in 2004. To date, a wide variety of 2D layered materials have been reported, which include: 1) monoelements: i) group III (borophene), ii) group IV (graphene and its derivatives such as GO, doped graphene, and graphane as well as silicone, germanene, and stanine), and iii) group V (phosphorene, 2D arsenic, antimony, and bismuth) elements; 2) metal chalcogenides: i) dichalcogenides with the formula MX_2_, where M consists of one or two transition metals from groups 4–10 (Ti, Hf, V, Nb, Re, Ta, Zr, Mo, W, Rh, Fe, Ni, Pd, Pt), X comprises one or two chalcogens (S, Se, Te); trichalcogenides (Bi_2_Te_3_, Sb_2_Te_3_, NbSe_3_, TaSe_3_); ii) group III (GaS, GaSe, InSe) and IV (SnSe, SnS, GeS, GeSe) monochalcogenides, and iii) others (SnS_2_); 3) metal oxides, such as PbO, MnO_2_, α‐MoO_3_, V_2_O_5_, and perovskites (LaNb_2_O_7_, LaMnO_3_); 4) metal hydroxides, such as *β‐*Ni(OH)_2_, layered double hydroxides (LDHs); 5) transition metal halides, such as PbI_2_, BiBr_2_; 6) nitrides, such as hexagonal boron nitride (*h*‐BN or white graphene), graphitic carbon nitride (*g*‐C_3_N_4_), and metal nitrides; 7) transition metal carbides; 8) transition metal hydrides; 9) transition metal phosphides (Li_7_MnP_4_, MnP_4_); 10) transition metal phosphates and phosphonates; 11) ternary bismuth tellurohalides BiTeX (X = I, Br, Cl); 12) MXenes derived from carbides and nitrides (M_n+1_AX_n_, [MAX] where *n* = 1 to 3, M is an early transition metal, A is an A‐group element [mostly IIIA and IVA, or groups 13 and 14] and X is either carbon, nitrogen, or both); 13) covalent organic frameworks.

These emerging 2D crystals range from insulators to semiconductors to semimetals and further to superconductors, thus providing utility for diverse applications. Note that there are still a large number of possible 2D materials (about 1700 compounds) that remain to be examined.^1^ Combining different 2D crystals enables the formation of lateral or van der Waals (vdW) (in one vertical stack) heterostructures exhibiting vertical stacking, affording exotic properties and functionalities due to charge transfers and synergistic effects between layers. Furthermore, 2D materials allow for alloying, doping, intercalation of ions and molecules, and chemical functionalization, offering flexibility in material design with tailored properties for expanding applications.^2^, ^3^, ^4^

### 1.2 Current Synthesis Methods

Two methodologies can produce 2D materials: 1) bottom‐up (atom by atom growth); and 2) top‐down (isolation from bulk) strategies. The former mainly include physical vapor deposition, (modified) chemical vapor deposition, molecular beam epitaxy, atomic layer epitaxy, growth on SiC (to make graphene), and wet chemical syntheses.^5^ Most of these methods require harsh reaction conditions (high temperature, high vacuum), rigorous control of substrate interactions, and complex post‐treatment steps (such as transfer and cleaning). Chemical synthesis can be performed at a relatively lower temperature (<200 °C) and allows better control at an atomic level. However, syntheses of large‐area 2D nanosheets by such bottom‐up routes need further development. Respectively, accompanying mechanisms underlying 2D crystal growth processes are still not sufficiently understood.

Top‐down approaches involve micromechanical cleavage (such as Scotch tape methods),^6^ anodic bonding (a variant of micromechanical cleavage),^7^ chemical exfoliation,^8^ and liquid‐phase exfoliation.^9^ Parent crystals suitable for exfoliation are composed of chemically bonded manifolds held together by weak vdW interactions with interlayer cohesive energies less than 130 meV Å^−2^.^1^ However, both micromechanical cleavage and anodic bonding are labor intensive with low yield and slow throughput. Also, they are not industrially scalable. Chemical exfoliation can be achieved either by alkali ion intercalation (Li^+^) followed by forced hydration (to release H_2_)^10^ or through oxidation using Brodie's, Staudenmaier's, or Hummers' methods or their variations. These routes have benefits of low cost and scalability. However, irreversible alteration of structure and properties occurs after chemical exfoliation. Additionally, interfering impurities originating from intercalants, oxidizing or reducing reagents may be covalently bound or strongly physisorbed to exfoliated flakes, complicating further processing. To circumvent these problems, direct exfoliation of layered materials in suitable liquids (solvents or co‐solvent mixtures, surfactant or polymer solutions, biomolecules, ionic liquids) appears to be a promising route for mass production of 2D nanosheets. This strategy is simple, flexible, versatile, readily scalable, and insensitive to environmental conditions. It also does not require expensive growth substrates. Chemically and colloidally stable flakes consisting of fewer than ten layers that can be obtained by such top‐down exfoliation can be further sorted and separated according to lateral dimensions and thickness via separation in centrifugal fields or in combination with density gradient ultracentrifugation. These suspension‐processable nanosheets allow for further blending, casting, and functionalization for a broad array of applications. Note that in some cases, a high yield of exfoliated flakes is obtained with accompanying flake scission and formation of defects and disorder. Quantification of such defects and disorder is an area of research offering important opportunity.

Ultrasonication‐assisted exfoliation is a simple method with proven scalability. However, unintended surface modification of nanosheets is introduced due to radicals formed by sonolysis in various solvents (water, alcohols, *N*‐methylpyrrolidone (NMP), and most solvent molecules). Hydroxyl radicals, HO·, tend to form in both oxygenated and deoxygenated water.^11^ Such hydroxyl radicals attack graphene's π system, for example. Basal plane carbon atoms can undergo addition reactions to produce covalent bonds, leading to hybridization transformations of them from *sp* ^2^ hybridization to *sp* ^3^ hybridization, with a slight out‐of‐plane puckering.^12^ Each surface hydroxyl group can coordinate up to three hydrogen‐bonded water molecules in such a way that can provide a satisfactory dispersion of graphene in pure water. This scenario is reminiscent of dissolution of graphene in chlorosulfonic acid owing to reactions of chlorosulfonic acid with conjugated rings resulting in covalent additions of sulfonate groups to graphenic six‐rings.^13^ NMP radicals resulting from sonolysis have also been demonstrated to contribute to MoS_2_ exfoliation.^14^

### 1.3 Applicability of SCFs in Processing 2D Materials

**Figure 1:**
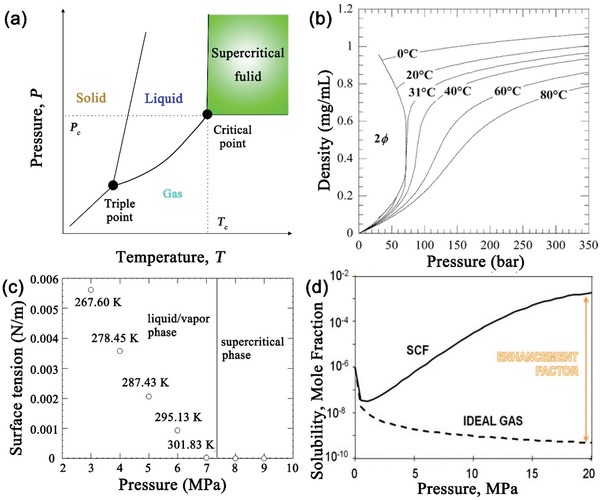
a) Schematic of a pressure–temperature phase diagram showing a triple point, a critical point, and a supercritical region. b) Carbon dioxide density versus pressure and temperature. Reproduced with permission.^16^ Copyright 2004, American Chemical Society. c) Surface tension of liquid CO_2_ versus pressure. These data points are labeled with their respective saturation temperatures at specific pressures. In a two‐phase region, the surface tension of a condensed phase decreases when its state approaches supercritical conditions. In a supercritical phase region, surface tension vanishes. Reproduced with permission.^19^ Copyright 2012, American Chemical Society. d) Solubility enhancement with SCF over ideal gas. Reproduced with permission.^20^ Copyright 2014, Royal Society of Chemistry.

An SCF is a substance at conditions above its critical temperature (*T*_c_) and pressure (*P*_c_), which was first recognized by Baron Charles Cagniard de la Tour in 1822. **Figure** **1**a shows a generalized temperature–pressure phase diagram which illustrates how *T*_c_ and *P*_c_ define the supercritical region. SCFs have both vapor‐ and liquid‐like physicochemical properties, such as near zero surface tension, low viscosity, high diffusion coefficients, excellent wetting of surfaces, and strong solvating power (Figure 1b–d).^15^, ^16^ The density of an SCF is about two orders of magnitude higher than that of its corresponding gas state, albeit less than half that of a conventional liquid (**Table** **1**).^17^ Viscosity and diffusivity are temperature and pressure dependent and are approximately an order of magnitude lower and higher, respectively, compared to a sub‐critical liquid. We emphasize that the physical properties of SCFs such as density, viscosity, and dielectric constant are tunable by altering temperature, pressure, or both, or addition of a co‐solvent. Figure 1d shows that typical solubilities in an SCF and an ideal gas differ. Solubilities of solutes (up to nearly six orders of magnitude) in SCFs are substantially enhanced in comparison with an ideal gas. These features make SCFs useful for both chemical reactions and materials synthesis that are impossible or difficult to be realized using traditional solvents. Also, using SCFs appears to apply to current industrial processing practices and is less damaging to equipment and contacting‐material structure.

**Table 1:** Comparison of selected properties of SCFs to those of liquids and gases

	Gas	SCF	Liquid
Density, ρ [g cm^3^]	10^−3^	0.1–1.0	1.0
Dynamic viscosity, *g* [mPa s]	0.01–0.3	0.01–0.03	0.2–3
Kinematic viscosity, *m* [10^−6^ m^2^ s^−1^]	5–500	0.02–0.1	0.1–5
Diffusivity, *D* [cm^2^ s ^−1^]	10^−1^	10^−3^	10^−5^
Thermal conductivity, λ × 10^3^ [W m^−1^ K^−1^]	4–30	28–80	80–250
Surface tension, σ [dyn cm^−1^]	0	0	20–50

### 1.4 Overview of Available SCFs

Common SCFs that are considered for processing of 2D materials are listed in **Table** **2**, along with their critical temperatures, pressures, and densities. SCFs that are gaseous at ambient conditions offer an advantage of evaporation after decompression to ambient conditions. This aspect obviates any need for drying and results in minimal solvent residues on nanosheet surfaces.

**Table 2:** Critical data for some fluids that may be useful in processing 2D materials

Fluid	*T*_c_ [°C]	*P*_c_ [MPa]^a)^	ρ_c_ [g cm^−3^]	Ref.
N_2_	−147.0	3.4	0.3109	21
CH_4_	−82.6	4.6	0.16	22
C_2_H_4_	9.5	5.1	0.218	23
CO_2_	31.0	7.4	0.469	17
C_2_H_6_	32.5	4.9	0.203	17
C_3_H_6_	91.9	4.62	0.23	23
Chlorodifluoromethane	96.0	4.9	0.524	24
C_3_H_8_	96.8	4.3	0.217	24
NH_3_	133.0	11.4	0.244	17
*n*‐C_4_H_10_	152.0	3.7	0.228	25
Diethyl ether	194.3	3.6	0.193	26
C_5_H_12_	197.0	3.4	0.237	23
C_6_H_14_	234.1	3.0	0.233	17
2‐PrOH, IPA (2‐propanol)	234.9	4.8	0.273	17
Acetone	235.0	4.8	0.269	19
Dichloromethane	236.7	6.1	0.440	27
MeOH	239.5	8.1	0.272	17
EtOH	240.9	6.1	0.276	17
Chloroform	261.8	5.3	0.491	28
1‐PrOH (1‐propanol)	263.5	5.2	0.275	17
Acetonitrile (ACN)	272.3	4.8	0.225	17
*t*‐BuOH (*t*‐butanol)	274.6	4.3	0.272	29
CCl_4_	283.3	4.5	0.557	17
Benzene	288.9	4.9	0.302	30
1‐BuOH (1‐butanol)	289.3	4.4	0.270	17
Toluene	319.0	4.1	0.292	23
*p*‐xylene (PX)	343.0	3.5	0.286	31
H_2_O	374.0	22.1	0.322	17
*N*,*N*‐dimethylformamide (DMF)	376.5	4.4	0.293	17
*N*‐methyl‐2‐pyrrolidone (NMP)	450.9	4.8	0.318	17

^a)^ 1 MPa = 10 bar.

ScCO_2_ is a well‐known SCF with a readily accessible critical point (Table 2). It has been used for exfoliating 2D crystals^18^ because it is cheap, abundant, nontoxic, nonflammable, recyclable, and environmentally friendly. ScCO_2_ can provide a repulsive free energy barrier to inhibit exfoliated sheets from re‐stacking, affording good stability while maintained in a supercritical state. However, scCO_2_ alone usually produces thick sheets (more than ten layers), though such thicknesses still offer advantageous applications. This limitation in exfoliation is probably due to CO_2_'s nonpolar nature and reversible and rapid escape after insertion into bulk layers on depressurization. Despite this limitation, flake thicknesses can be reduced further by cycling into and out of supercritical states.^32^, ^33^, ^34^ Alternatively, exfoliation of layered structures can be extended and accelerated by using scCO_2_ mixed with various solvents.^35^

ScN_2_ (supercritical nitrogen) is another SCF in which exfoliation of graphene^21^ and clays^36^ has been done. Yields and detailed characterizations of such exfoliated sheets have not been extensively reported.

ScNH_3_ (supercritical ammonia) has been used for exfoliation of natural graphite.^37^ A product yield of ≈3 wt% was reported, and about 40% of exfoliated sheets had less than five layers upon treatment (200 °C, 15 MPa) for 1 h. Lateral dimensions ranged from 5 to 8 µm, in contrast to less than 3 µm reported in most liquid‐phase exfoliation methods. Interestingly, scNH_3_ also provides simultaneous nitrogen doping of graphene layers during exfoliation, which suggests an intrinsic chemical modification.

ScCHClF_2_ (supercritical chlorodifluoromethane)^24^ and an scCO_2_‐CH_2_Cl_2_ (dichloromethane)^27^ mixture can assist in exfoliation of fluorinated clays and promote good dispersion of exfoliated clays in polymer matrixes for the preparation of polymer/clay nanocomposites. Perfluorinated polymers and surfactants are often found to be soluble in scCO_2_ and offer several advantages when designing scCO_2_‐based processing procedures.

Other solvents that are SCFs at mild conditions include C_2_H_4_, C_2_H_6_, and C_3_H_8_. These SCFs need to be investigated further for their efficacy in intercalating and exfoliating 2D materials.

Some claims are occasionally made asserting supercritical processing where the stated conditions correspond to subcritical or near critical physical states. In such cases, we refer to such near critical conditions, for example, as applied to NMP: ncNMP; scNMP conditions are: *T*_c_ ≈ 450.9 °C, *P*_c_ ≈ 4.8 MPa (Table 2). It is also important to note that many mixed gas and liquid systems are investigated, wherein criticality is claimed, but authoritative references or experimentation are not provided to confirm supercriticality for a given mixture. We attempt to distinguish these cases for readers when they arise in our discussions below.

### 1.5 Initial Exfoliation Applications

**Figure 2:**
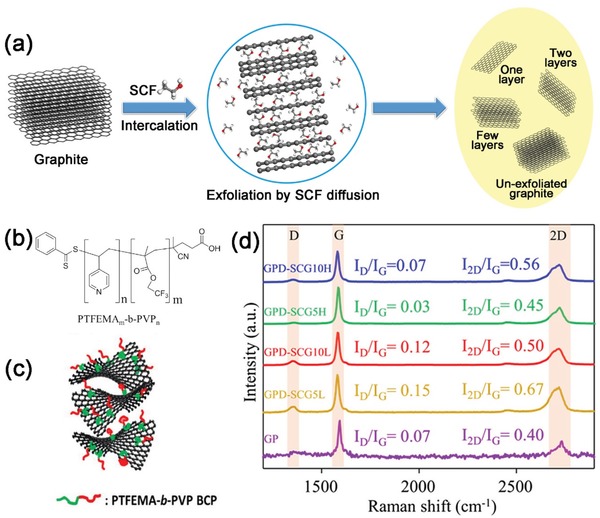
a) Schematic of SCF exfoliation of graphite crystals into few‐layer graphene. b) Molecular structure of PTFEMA‐b‐PVP amphiphilic fluorinated block copolymer. c) Schematic of PTFEMA‐b‐PVP stabilized graphene exfoliated in scCO_2_. d) Raman spectra for precipitates of graphite nanoplatelet (GP) and exfoliated graphene samples stabilized by PTFEMA‐b‐PVP copolymers with low (L) and high (H) molecular weights. Sample ID digits indicate the number in weight percentages of block copolymer. Reproduced with permission.^40^ Copyright 2017, Elsevier Inc.

Since Gulari et al. demonstrated the first intercalation of layered silicates by using supercritical CO_2_, scCO_2_, as a processing medium,^38^ extensive efforts have been devoted to intercalating and exfoliating layered 2D materials in various SCFs to yield single‐ and few‐layer nanosheets (**Figure** **2**a, the SCF intercalation and expansion pictured are discussed at length later). SCFs possess easily tunable solvation strength and favorable interfacial tensions, wetting, and transport properties, making them unique and potentially superior solvent media for rapid exfoliation of 2D nanosheets.^39^ Compared with liquid‐phase exfoliation via ultrasonication, an SCF exfoliation strategy requires shorter processing time and tends to produce highly crystalline single and few layers with lower defect levels and larger flake sizes. Indeed, Raman data have indicated that D‐band to G‐band intensity ratios (*I*_D_/*I*_G_) are ‘‘sufficiently low'' of only 0.03 for stabilized graphene by poly(2,2,2‐trifluoroethyl methacrylate)‐*block*‐poly(4‐vinylpyridine) in scCO_2_ (Figure 2b–d).^40^ Fluorinated polymers and surfactants are well‐established stabilizers for use in scCO_2_‐based dispersion, suspensions, and complex fluids.^41^

These *I*_D_/*I*_G_ ratios are important because they are a common metric for graphene “quality” or “pristineness.” The Raman scattering intensities at 1330 to 1370 cm^–1^ and 1540 to 1590 cm^−1^,^42^, ^43^, ^44^ respectively, are ascribed to D‐band and G‐band. This D transition in pristine graphene is forbidden (has zero intensity),^45^ but ratios up to 0.3 have been claimed to demonstrate “pristine” character. Therefore, one must be critical in evaluating claims of quality based on Raman measurements. When defects are introduced into graphene sheets by chemical reactions that disrupt the aromatic 2p_z_ electron density of individual carbon atoms, carbon–carbon bonds are distorted from planarity, and the carbon centers associated with such defects acquire *sp* ^3^ hybridization. Such chemical effects result in increasing *I*_D_ intensity. The G‐band is due to C—C bond stretching and is an allowed Raman transition in pristine graphene, and its intensity, *I*_G_, is used to “normalize” D‐band intensity, via the *I*_D_/*I*_G_ ratio. The so‐called 2D band is a two‐phonon band whose shape and position depend somewhat on excitation wavelength. Its intensity variation has not yet succumbed to a simple structural interpretation useful for quantifying defect formation in graphene.

Particularly, in comparison to liquid‐phase exfoliation that often, unfortunately, relies on centrifugation and generates high waste, SCF technology does not rely on centrifugation. Avoiding centrifugation is attractive to extant dispersion manufacturing processes. We should note that a direct comparison of SCF exfoliated sheets and those obtained by liquid‐phase exfoliation remains an issue because of differences in analysis techniques and procedures, initial bulk concentration, and how raw materials and layered materials are obtained. Also, some workers include centrifugation processing in their SCF exfoliation studies, conflating exfoliation and sedimentation effects. Some care must be exercised in reviewing exfoliation claims obtained by SCF processing, in order to ascertain whether or not centrifugation has also been included in the processing described.

SCFs are proving advantageous for processing and functionalization of 2D materials. However, SCF approaches have received less attention than expected. Only two earlier reviews summarize exfoliation and processing of graphene in SCFs.^17^, ^46^ Discussions of supercritical processing of many other 2D crystalline materials undergoing rapid application development are lacking. Roles played by SCFs in functionalization, phase transformation, and composite formation of 2D materials remain to be systematically examined. We focus on these aspects in this progress report and also highlight some fundamentals underlying SCF technologies to gain insight for optimization of SCF conditions. Further, applications of 2D materials and composites derived by SCF processing are summarized. We also describe prospects for future developments for processing 2D materials in SCFs.

## 2 SCF‐Assisted Processes

Along with progress in understanding associated phase equilibria and fluid properties, various processes for using SCFs are being developed for 2D materials. In this section, we briefly discuss six SCF‐based processing methods that have been applied to 2D inorganic crystals and hybrids. All of these techniques have previously been applied in other aspects of particle technology, and are beginning to be used with layered materials.

### 2.1 Rapid Expansion from Supercritical Suspensions

**Figure 3:**
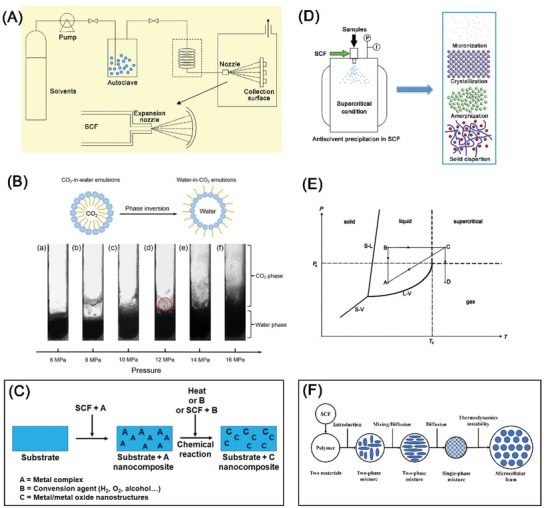
A) Schematic of rapid expansion from supercritical suspensions. Reproduced with permission.^47^ Copyright 2014, Royal Society of Chemistry. See text for expanded discussion. B) Phase behavior of emulsion in CO_2_/PVP/H_2_O system (upper part) and photographs of the system with different CO_2_ pressures (lower part). Reproduced under the terms of the Creative Commons Attribution 4.0 International License.^48^ Copyright 2015, the Authors, Published by Springer Nature. C) Schematic illustration of supercritical fluid chemical deposition. D) Schematic of antisolvent precipitation in SCF. E) Schematic representation of supercritical drying: The solvent is pressurized and heated beyond the critical point (*P*_c_, *T*_c_) via one of two pathways either path ABC or path AC. This is followed by depressurization (path CD). In this way, the interface of gas and liquid is circumvented. S−L, L−V, and S−V denote solid−liquid, liquid−vapor, and solid−vapor equilibrium curves, respectively. Reproduced with permission.^19^ Copyright 2012, American Chemical Society. F) Schematic of supercritical foaming.

Traditional rapid expansion from supercritical suspension (RESS) processes were used for producing particles as a result of precipitation of dissolved materials at a very high rate of supersaturation due to sudden expansion upon depressurization. This process was also applied to exfoliate layered materials (**Figure** **3**A).^49^ The RESS process illustrated in Figure 3A involves an SCF and an optional co‐solvent.^47^ On pressurization to a supercritical state, the layered material is enveloped by SCF and intercalated by SCF and cosolvent. Depressurization leads to expansion and layer separation due to pressure gradients, and the SCF reverts to a subcritical state.

Gulari and coworkers first reported an scCO_2_‐processing technique for intercalating and exfoliating layered silicates^38^ and graphite.^50^ Following this pioneering work, a variety of 2D crystals have been exfoliated using similar or modified SCF technology, including graphene,^17^, ^47^ fluorographene,^51^ BN,^52^, ^53^, ^54^ TMDs (MoS_2_, WS_2_, MoSe_2_, etc.),^52^, ^55^, ^56^, ^57^, ^58^ black phosphorus,^59^ NH_4_MPO_4_ (M = Fe, Mn, Co, Ni),^60^ titanate,^61^, ^62^ V_2_O_5_,^63^ MoO_3_,^64^, ^65^ Mn_3_O_4_,^66^ 2H‐WS_2_/2H‐MoS_2_,^67^ and BN‐MoS_2_ heterostructures,^39^ among others. In SCF‐assisted exfoliation, a layered material is initially added to a solvent or solvent mixture and then sealed in a high‐pressure reactor. Temperature and pressure are increased to drive any solvent to a critical state. A good solvent intercalates between nanosheet layers under critical conditions. This intercalation causes expansion of the layered material. After incubation for a while, a high‐pressure reactor is abruptly depressurized to induce expansion. This expansion causes large pressure gradients between material interlayer regions and ambient conditions, and these gradients push layers apart with formation of mono‐, few‐, and multi‐layer sheets.

To promote SCF intercalation and exfoliation, methods such as stirring,^40^ ultrasonication (cavitation),^32^, ^68^, ^69^ mechanical shear mixing by a rotor stator,^53^, ^70^, ^71^, ^72^ or ball milling^73^ have been used. These mechanical and sonochemical methods may or may not also induce edge or basal plane surface modification chemistry that might aid exfoliation. Addition of co‐solvent during an SCF process, such as NMP,^72^ or a stabilizer such as pyrene and pyrene‐derivatives,^74^, ^75^ or poly(2,2,2‐trifluoroethyl methacrylate)‐*block*‐poly(4‐vinylpyridine)^40^ can enhance interactions with nanosheet surfaces and further improve exfoliation efficiency. Coupling stirring, rotor‐stator, and ultrasonication approaches to providing shear along with SCF depressurization, while such multiphase mixtures are in a supercritical state, appear to provide synergistic effects. Some reports of sequential processing, with an SCF process followed by a sub‐critical mechanical shear or ultrasonication process,^48^, ^72^, ^76^ often do not offer real advantages and rely upon nanosheet selection by centrifugation to obtain useful but very dilute samples. Moreover, when auxiliary chemomechanical processing steps are included (high shear, ultrasonication), one must be very circumspect in assigning causality in any hypothesized exfoliation mechanistic discussion. Presumably, SCF intercalation can greatly facilitate shear‐induced exfoliation prior to reductions in pressure and loss of criticality.

### 2.2 Complex Fluid Processing

CO_2_‐in‐water emulsions and microemulsions stabilized by PFPE, a perfluoropolyether, are formed at a relatively low CO_2_ pressure (8 MPa), while reverse emulsions and microemulsions of water‐in‐CO_2_ are induced at elevated CO_2_ pressure (>12 MPa) as shown in Figure 3B.^77^ ScCO_2_ serves as a “switch” to tune surfactant molecular aggregation, which is reversible and can be realized by pressurization and depressurization. ScCO_2_ pressure variations tune such microemulsion–reverse microemulsion continuous phase transitions.

A microemulsion‐based process has been claimed to facilitate exfoliation of layered materials.^48^, ^76^, ^78^, ^79^, ^80^ Polyvinylpyrrolidone (PVP) outperformed Pluronics F127 and P123, Tween 20, hexadecyltrimethylammonium bromide (CTAB), and sodium dodecylbenzenesulfonate (SDBS) for exfoliation in scCO_2_ mixed solvent solutions.^76^ The pyrrolidone groups of PVP have a strong affinity for nanosheet surfaces in these layered materials, providing stabilization against re‐aggregation. However, no evidence of microemulsion formation was provided, and an actual process described more closely resembled aqueous ethanol ultrasonication followed by centrifugation and afforded meager yields. After discarding most of the graphene mass, the sediment, remaining dispersion concentrations obtained were less than 0.2% by weight^76^ and comprised graphene that exhibited an optical absorption coefficient less than 20% that of pristine graphene.

Water‐in‐CO_2_ microemulsions in scCO_2_ also provide unique media for uniform decoration of 2D materials with polymer,^81^ metal, or metal oxide particles. Aqueous micelle cores with nanometer dimensions allow for solubility of precursors (such as metal salts that are insoluble in CO_2_) and act as nanoreactors to compartmentalize precipitation.^82^, ^83^, ^84^, ^85^ In microemulsions, there is no phase boundary between co‐dissolved immiscible liquids. It is challenging to capture nanoscale dimensions of metal and metal oxide nanoparticles in reverse microemulsions unless one can promote these nanoparticle formation kinetics to make them competitive with surfactant reorganization kinetics in swollen microemulsion systems.^86^ Interfaces of scCO_2_ and water can also be used to synthesize hollow oxides (such as silica, titania).^87^

### 2.3 Supercritical Fluid Chemical Deposition

Supercritical fluid chemical deposition (SFCD) techniques allow one to deposit particles and films (Figure 3C),^88^, ^89^, ^90^ including metal nanoparticles in polymer matrices^91^ and in alumina membrane pores,^92^ conformal Pd films on Si and polyimide substrates,^93^ ruthenium nanoparticles on carbon nanotube (CNT) surfaces,^94^ and ZrO_2_ films on CNTs.^95^ Compared to conventional solvents, SCFs can promote conformal coverage of complex surfaces and poorly wettable substrates (such as graphene or other 2D materials) with dissolved precursors (such as chelate or metal β‐diketone complexes) more readily. After reaction at specific temperature and pressure, the formed species from adsorbed compounds tend to nucleate, grow, and deposit on any available support.^96^, ^97^, ^98^, ^99^, ^100^, ^101^, ^102^, ^103^, ^104^, ^105^, ^106^

These SFCD processes can overcome obstacles of high interfacial tension and viscosity in graphene oxide colloids, which are challenging for most existing methods. Porous graphene decorated with multimetallic nanoparticles (NPs) with small size and tunable loading has been fabricated in scCO_2_.^107^ Some co‐solvents such as methanol, ethanol, or tetrahydrofuran can be employed to modify the polarity of CO_2_ and enhance dissolution of precursors in scCO_2_.^107^, ^108^ Mechanistically, an SCF wets both substrate and particle materials and makes it feasible to form uniformly distributed dispersions at SC conditions and to then deposit these materials as pressure and temperature are decreased.

### 2.4 Antisolvent Precipitation in SCFs

This supercritical technique involves a mixed solvent system with a solute compound dissolved in an organic solvent but insoluble in the SCF or with solute dissolved in an SCF but insoluble in an organic solvent, where the organic solvent and SCF are miscible. When a solvent, acting as an antisolvent, is combined with scCO_2_ in which a solute is soluble, this mixture becomes supersaturated.^109^ This saturation induces nucleation of small particles and their precipitation when expanded into an antisolvent as illustrated in Figure 3D. This supercritical antisolvent strategy offers a route for preparing 2D material composites.^110^, ^111^, ^112^

Conversely, as scCO_2_ is introduced into a solvent dispersion containing few‐layer nanosheets and a solute compound soluble in this solvent, solute solubility decreases if it is not soluble in scCO_2_, causing supersaturation and particle condensation.^113^, ^114^ This process routinely uses the SCF to also extract organic solvents for production of dry particle powders useful in pharma. However, this saturation may also lead to nucleation of nanoparticles and their deposition onto nanosheet surfaces. A further chemical or physical treatment rendering such nanoparticles insoluble in the solvent would provide an alternative pathway to a 2D material composite. However, absent such a chemical passivation step, such particles would be expected to redissolve in the solvent.

Both of these approaches use an SCF to drive exfoliation during and after precipitation of particles. However, while both approaches have been used to make particles for various applications, they have not yet been applied to make particle composites with 2D materials.

### 2.5 Supercritical Drying

Supercritical drying provides advantages for processing of 2D materials and hybrids, such as drying of graphene‐based aerogels.^115^, ^116^, ^117^ Conventional solvent evaporation by heating leads to large capillary forces and causes microstructure collapse, resulting in low specific surface areas. This type of problem can be solved by using a supercritical drying protocol. Meanwhile, supercritical drying can avoid the formation of any liquid–gas interface, resulting in smaller particles with increased specific surface areas and higher homogeneity (Figure 3E).

### 2.6 Supercritical Foaming

Polymer foams or scaffolds can be obtained by using SCF as a physical blowing agent, provided there exists a sufficient pressure‐dependent solubility of the blowing agent in the polymeric prefoam (Figure 3F). This scheme can be applied to make 2D material‐based porous polymer composites.^17^, ^118^, ^119^, ^120^, ^121^, ^122^, ^123^, ^124^, ^125^ Specifically, 2D crystal–polymer composites are first swollen and plasticized with dissolved SCF (such as scCO_2_) for some periods. After rapid pressure reduction, CO_2_ solubility in a composite decreases, causing nucleation and growth of oversaturated CO_2_ to generate bubbles. As a result, 2D nanosheets coated with a porous polymer network are formed. Such porous structures encompass closed cell pores, open‐cell pores (interconnected cells), macropores, and mesopores. Foam inner morphology relies on SCF saturation level that is associated with several parameters such as SCF pressure and temperature, dynamics of nucleation and bubble growth (due to pressure drop and interfacial stabilization by nanosheets^126^), and SCF pressure reduction rate. Subcritical CO_2_ has been used to affect graphene exfoliation in polypropylene (PP)/G composites.^123^

Graphene nanoplatelets (GNP) were mixed with high‐density polyethylene (HDPE) in a tandem extruder assembly at about 1.1% by volume. CO_2_ was used as a blowing agent and SCF to produce foams having a high permittivity and low dielectric loss.^125^ The GNP had nominal dimensions of 50 µm in diameter and 20 nm in thickness. A temperature range of 130–215 °C in this extrusion system resulted in good dispersion and partial exfoliation. CO_2_ was injected into the first extruder and dissolved under thermal and pressure conditions therein. When exiting a second extruder, a drop in pressure and temperature resulted in foaming to produce a nanocomposite with a permittivity, ε′, of about 77.5 and a dielectric loss, ε″, of about 0.233 at 10^5^ Hz.^125^ This foaming action resulted in a positive synergistic effect to produce parallel GNPs that were very polarizable in applied fields. A low density of about 0.15 g cm^−3^ that is very difficult to achieve when formulating high dielectric inorganic nanoparticles (e.g., BaTiO_3_) in polymer matrices is another positive attribute of this advanced dielectric material.

Polyvinyl alcohol/GO nanocomposite foams have been evaluated using scCO_2_.^122^, ^124^ In one study, pan milling to prepare a PVA/GO nanocomposite was followed by melt extrusion with varying amounts of PVA, followed by scCO_2_‐based blowing to create foam materials at various temperatures and depressurization rates.^124^ A foam prepared at 80 °C containing 2.5% GO resulted in pores of 17.9 µm in average diameter compared to 29.1 µm for a PVA‐only control. This GO nanocomposite in compression to 50% strain exhibited a maximum stress of 178% relative to that of the PVA‐only control. These improved mechanical properties and decreased pore diameters were attributed to the incorporation of GO in the pore walls.^124^ In a related process, aqueous PVA/GO dispersions were partially dried to produce hydrated mixtures and then compression molded into thin plates at 165 °C.^122^ These plates were then infused with scCO_2_ at 100 °C and 15 MPa followed by decreasing the pressure at 5 MPa s^−1^. GO contents of 0%, 0.1%, 0.5%, and 2.5% showed that pore diameter decreased and compressive strength increased with increasing GO content to 0.5%; the 2.5% GO content showed no improvement over the 0.5% GO level.^122^ A significant physical aspect of these studies is the stabilization of smaller pores by the GO sheets. This stabilization can be understood in terms of a Pickering stabilization effect as recently reviewed for G and GO.^126^

Another useful material was obtained by using scN_2_ to induce exfoliation and foaming in graphene mixtures with HDPE.^127^ Foaming degrees of 7%, 16%, and 26% were obtained, and a transient plane source hot disk thermal constant analyzer was used for conductivity analysis. These analyses yielded a solid‐phase conductivity of 4 W m^−1^ K^−1^ according to a Maxwell–Eucken model.^127^ Moderate foaming (7%) gave the highest bulk conductivity that increased with GNP from 0.8 W m^−1^ K^−1^ (4.5 vol%) to 3.75 W m^−1^ K^−1^ (18 vol%). Further increases in foaming decreased bulk conductivity.

## 3 Exfoliation and Chemical Processing in SCFs

We discuss features of critical solvent parameters and other orthogonal processes that affect exfoliation, which appear essential for using SCF‐based and SCF‐assisted processes. It is always appropriate to consider how additives, co‐solvents, and mechanicochemical treatments may impact nanosheet surface chemistries.

### 3.1 Exfoliation Facilitated by SCFs

Before initiating exfoliation, SCFs are considered to first intercalate into galleries or nanosheet interlayers to expand interlayer separation and swell these materials. Increasing pressure can promote such SCF intercalation and expansion of spaces between interlayers.^128^ Increasing pressure results in a higher free energy barrier and reduced interlayer attraction, which also enhances colloidal stability of exfoliated sheets in dispersion.^129^ Two parameters are critical to this process: 1) any pretreatment of bulk materials; and 2) SCF type. Using pretreated sample such as surfactant (e.g., alkyl‐based quaternary ammonium surfactants)‐modified natural clays^38^, ^130^ or nitric acid–treated graphite^131^, ^132^ as a starting material facilitates nanosheet‐solvent interaction as well as intercalation of SCF molecules and expansion of these interlayer gallery spacings due to physical and chemical modifications. As a consequence, exfoliated sheet yields are improved. However, a significant increase in exfoliation efficiency by oxidation pretreatments is often accompanied by a degradation in a sample's quality. These effects need to be considered when designing further processing and applications. A summary of 2D materials exfoliated in various SCFs is provided in **Table** **3**.

**Table 3:** Summary of 2D materials exfoliated in SCFs

2D materials	SCF	Exfoliation condition	Yield	Dimension	Ref.
Silicate	NcCO_2_,^a)^ ScCO_2_–sugar acetate^a)^	11.7 MPa, 40 °C, 24 h (dense gas solution, DGS); 7.9 MPa, 40 °C, 24 h (gas‐expanded liquid, GEL)	N/A	Thickness: 3.2 layers (DGS); 2.9 layers (GEL)	38
Graphene	ScCO_2_	10 MPa, 45 °C, 30 min	≈30 to 40 wt%	Thickness: ≈10 layers Lateral size: a few micrometers	33
Graphene	ScCO_2_	8 MPa, 40 °C under ultrasonication (0, 60, 120, or 300 W), 30 min, then CF: 1500 rpm, 60 min	N/A	Thickness: 3.6–7.2 nm, 1.6–3.2 nm, 0.79–0.86 nm, 0.44–0.61 nm Lateral size: 5–100 µm, 2–15 µm, 0.5–10 µm, 50–100 nm (corresponding to 0, 60, 120, and 300 W, respectively)	32
Graphene	ScCO_2_	12 MPa, 40 °C under ultrasonication (120 W), 60 min	16.7 wt%	Thickness: 1−3 layers Lateral size: 0.5−5.0 µm	128
Graphene	NcNMP,^a)^ ScNMP/scEtOH, and scDMF	Ultrasonication (160 W): 10 min, then 38–40 MPa, 300–400 °C, 3 min	N/A	Thickness: 1–10 layers Lateral size: 0.1–2 µm	133
Graphene	ScDMF	Ultrasonication (180 W): 15 min, then 400 °C, 15 min	3.9 wt%	Thickness: 0.72 nm	132
Graphene	ScDMF	Ultrasonication (180 W): 15 min, then 400 °C, 15 min	7 wt%	Thickness: 3 nm Lateral size: 2–10 µm	131
Graphene	ScCO_2_	15 MPa, 45 °C, 30 min	N/A	Thickness: 1.0–6.0 nm Lateral size: 0.2–1.0 µm	34
Graphene	ScH_2_O	500 °C, 10 min	N/A	N/A	134
Graphene	ScEtOH	400 °C, 10 min	N/A	Thickness: <3 nm Lateral size: <30 nm	135
Graphene	ScCO_2_	Ultrasonication (120 W): 3 h, then 16 MPa, 40 °C, 6 h, and ultrasonication (120 W): 2 h, CF: 9000 rpm, 20 min	51.8 wt%	Thickness: <5 layers	136
Graphene	ScCO_2_	Ultrasonication: 2 h; 16 MPa, 40 °C, 3 h; then ultrasonication: 3 h, CF: 9000 rpm, 15 min	N/A	Lateral size: 0.5–20 µm	137
Fluorographene	ScCO_2_–glycol^a)^	10 MPa, 50 °C, 24 h, then magnetic stirring, 1 h and ultrasonication (250 W), 30 min in ethanol/water solution	32 wt%	Presence of monolayer	51
Reduced graphene (rGO)	ScEtOH/scMeOH/scPrOH and scBuOH	Ultrasonication: 1 h; 12.9–36.5 MPa, 400 °C, 2 h	N/A	N/A	29
N doped graphene	ScACN	310 °C, 2–24 h	N/A	N/A	138
N‐doped graphene	ScNH_3_	15 MPa, 200 °C, 1–2 h, then CF: 1000 rpm, thrice	≈3 wt%	Thickness: <5 layers (40%) Lateral size: 5–8 µm	37
BN	ScCO_2_	10 MPa, 45 °C under ultrasonication (60 W), 40 min	N/A	Thickness: <5 layers (90%), 1 layer (20%), 2 layers (40%) Lateral sizes: 0.5−2 µm	52
MoS_2_, MoSe_2_	ScDMF	Ultrasonication (160 W): 5 min, then 400 °C, 1 h, CF: 2000 rpm, 30 min and 30000 rpm, 1 h	N/A	Thickness: 4–5 layers (35–40%) Lateral sizes: 0.5 to 1 µm	56
MoS_2_	ScCO_2_	16 MPa, 75 °C under shearing (1200 rpm), 3 h, then ultrasonication, 30 min in ethanol, CF: 2000 rpm, 30 min	N/A	Thickness: <10 layers (95%), 1–4 layers (50%)	57
BP	ScCO_2_–NMP	Ultrasonication (150 W): 1 h, then 15 MPa, 40 °C, 3 h, and ultrasonication: 1 h	N/A	Thickness: predominantly 3–5 layers	59
LiFePO_4_/C, LiMnPO_4_/C, LiCoPO_4_/C, LiNiPO_4_/C	ScEtOH–PVP	10 MPa, 400 °C, 2 h	N/A	Thickness: 4.3, 1.6, 2.1, and 1.7 nm Lateral size: 0.5, 0.5–1.5, ≈0.5, and 0.5–1.0 µm (corresponding to LiFePO_4_/C, LiMnPO_4_/C, LiCoPO_4_/C, LiNiPO_4_/C, respectively)	60
Titania	ScDMF	400 °C, 15 min, CF: 3000 rpm, 3 min, then 8000 rpm, 20 min	N/A	Thickness: 1 layer (≈8%)	61
V_2_O_5_	ScEtOH	250 °C, 12 h	N/A	Thickness: ≈6 nm	63
Amorphous MoO_3_	ScCO_2_–ethanol/water^a)^	Ultrasonication: 60 min, then 16 MPa, 80 °C, 3 h, CF: 6000 rpm, 15 min	N/A	Thicknesses: 3–4 nm (2–3 layers) Lateral size: 140 nm	65
Mn_3_O_4_	ScDMF	Ultrasonication: 10 min, then 400 °C followed by rapid cooling	N/A	Thicknesses: nanoscale Lateral size: hundreds of nm	66

^a)^ Definitive statement or evidence of supercriticality absent.

A fundamental aspect of SCF‐assisted exfoliation is that 1) intercalation of SCFs and often other solvents, polymers, and stabilizers occurs under supercritical conditions, and 2) when high pressure is released, expansion of SCF within interlayers drives sheets apart to yield exfoliation that is stabilized or not, depending on the conditions and environment of pressure release. Gulari and Serjatkulu^50^ showed in their early studies of graphite exfoliation using PDMS solutions in scCO_2_ that X‐ray powder diffraction peaks at 2θ values over 26° to 27° were significantly attenuated and broadened as a result of such intercalation and expansion. Similarly, intersheet spacings in layered silica (Na^+^ Cloisite) were shown to substantially expand on scCO_2_ treatment with galactose‐based stabilizers.^38^

While most reports of SCF‐driven exfoliation have not included corroborating X‐ray diffraction evidence that intercalation by SCFs and stabilizers when present occurs, past SCF expansion data unequivocally demonstrate that exfoliation has occurred. Exfoliation in the absence of intercalation can only occur if an external adhesive force is applied, or a sufficiently high shear force is applied. It can, therefore, be concluded that intercalation driven by SCF is a starting point for exfoliation, at least in processes without high shear fields or ultrasonication. It would be useful to quantify intercalation by specific SCFs, particularly in situ if possible. In view of diverse applications of powder X‐ray diffraction to particle and materials syntheses in SCFs,^139^, ^140^ such valuable studies are feasible.

#### 3.1.1 High‐Boiling Solvents

Efforts have been made to study interactions between solvent molecules and 2D materials, in searching for appropriate solvents for effective exfoliation. Exfoliation appears to occur when van der Waals forces that are sufficiently small between adjacent nanosheets are overcome.^141^ Texter claimed that the best solvents from a wetting perspective are those that satisfy the inequality: γ_2D_ > γ_sol_ + γ_2D/sol_, where γ_2D_ is the Gibbs surface energy of a 2D material, γ_sol_ is the surface energy (surface tension) of a solvent (a Gibbs free energy that can be readily measured), and γ_2D/sol_ is the interfacial energy of the solvent/2D material interface (that can be estimated from contact angle measurement).^142^ This inequality guarantees a positive spreading coefficient and spontaneous wetting by the solvent.

Over 70 solvents have been examined for exfoliation, and some apparently good solvents have been identified for various 2D crystals.^141^ However, only a few of these solvents such as high‐boiling‐point NMP and DMF have been employed under supercritical and near critical conditions for exfoliating graphite,^131^, ^132^, ^133^ layered titanate,^61^ NH_4_MPO_4_·H_2_O (M = Fe, Mn, Co, Ni),^60^ MoS_2_,^56^, ^143^ MoSe_2_,^56^ BN,^144^ and BN‐MoS_2_ heterostructure.^39^ Compared to gaseous molecules, such high‐boiling‐point solvents have a stronger affinity for surfaces of 2D materials. This affinity seemingly enables higher exfoliation efficiency and better stabilization to prevent re‐stacking. For instance, after a short processing time of 15 min in ncNMP (near critical NMP) and scDMF (supercritical DMF), natural graphite exfoliation into few‐layer sheets in which 90% to 95% are 10‐layer or thinner flakes and 5–9% are monolayers was claimed.^133^ Stable and dilute dispersions with a concentration up to 2–4 mg mL^−1^ were claimed. Such yields contrast with a very low (nearly zero) yield of monolayer graphene in both scCO_2_ and scNH_3_. However, both NMP and DMF unequivocally present processing challenges due to their high boiling points.

Mixed solvents such as NMP/water,^145^ NMP/*N*‐octylbenzene,^146^ and DMF/*N*‐butyl alcohol^147^ have been shown to enhance nanosheet yield through ultrasonication‐based exfoliation. Nevertheless, there are only a few reports on using such supercritical mixtures for exfoliation, and it seems unequivocal that ultrasonication of water‐containing mixtures produces hydroxyl radicals that undergo addition reactions with basal planes of graphite and graphene.^142^

#### 3.1.2 Low‐Boiling (Mixed) Solvents

In order to address issues of high cost, toxicology, and vapor pressure for some good solvents with high boiling points, using low‐boiling‐point solvents would be preferred, if sufficiently concentrated dispersions could be obtained. As an alternative, supercritical alcohols such as scEtOH (supercritical ethanol),^63^, ^133^, ^135^, ^148^ scACN (supercritical acetonitrile),^138^ and a mixture of scIPA (supercritical isopropanol)^54^ or scEtOH^148^ and water have been studied. These alternative solvents apparently provide sufficient penetration and intercalation to yield exfoliation of mono‐ and few‐layer sheets. Higher supercritical density provides a stronger repulsive free energy barrier to sheet re‐aggregation and, therefore, enhanced exfoliation efficiency.^129^, ^148^ Tuning the Hansen solubility parameters of SCFs by controlling process conditions was claimed to promote enhancement of exfoliation yield.^148^ Other potential low‐boiling solvents such as acetone, chloroform, and mixtures remain to be investigated for different 2D crystals by using SCF exfoliation processing.

#### 3.1.3 Cavitation and Shear Force–Assisted Exfoliation in SCFs

**Figure 4:**
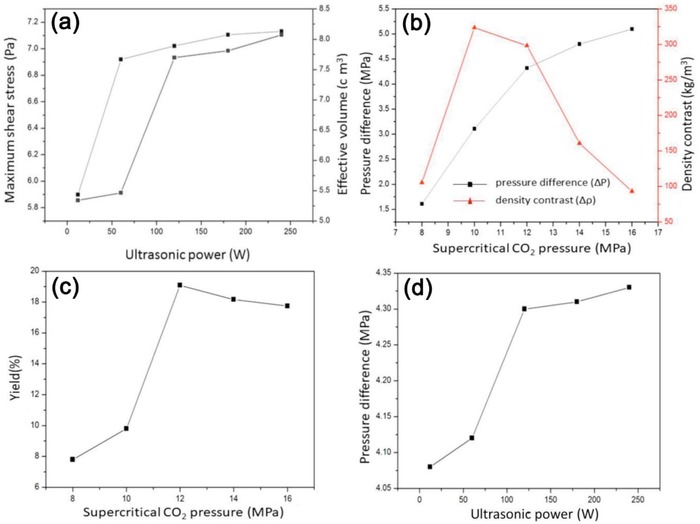
a) Variation of maximum shear stress and effective volume versus ultrasonic power at 12 MPa. b) Variation of pressure difference and density contrast, and c) yield of graphene versus CO_2_ pressure at 120 W. d) Variation of pressure difference versus ultrasonic power at 12 MPa. Reproduced with permission.^69^ Copyright 2017, Elsevier B.V.

Cavitation occurs during rarefaction cycles of ultrasound waves to create transient microbubbles, which collapse and form microjets and shock waves. Shock waves can break bulk flakes into thin layers and also cause scission in lateral dimensions. Meanwhile, microturbulence (or micro‐convection) and pitting effects stemming from bubble collapse provide mechanical energy (mostly in the form of tensile stress) to overcome attractive interactions between the layers, inducing exfoliation. Increasing fluid pressure was reported to decrease a cavitation threshold, thus suppressing any creation of cavitation bubbles. Nevertheless, a sufficiently large increase in applied ultrasound intensity could induce cavitation, even at high overpressures. A collapse of such transient cavities leads to a higher intensity and consequently enhanced sonolysis effects. Intensive cavitation in a pressurized reactor can substantially enhance exfoliation efficiency in RESS.^52^, ^128^, ^149^ Cavitation was claimed to be unlikely in scCO_2_ due to an absence of phase boundaries, but loading with bulk layered materials provides phase boundaries. Although insoluble impurities in scCO_2_ might serve as sites where nucleation of cavitation bubbles can take place, resulting cavitation intensity is believed to be small and not significant in aiding exfoliation. Under such circumstances, shear stress and pressure fluctuations generated by ultrasound were conjectured to be mainly responsible for enhanced exfoliation yields in scCO_2_.^69^ Computational fluid dynamics (CFD) simulation showed that the maximal shear stress along with the amount of active area increased with ultrasonic power from 12 to 240 W, which may contribute to graphite exfoliation in scCO_2_ (**Figure** **4**a). Meanwhile, exfoliation yield was linked to differences of pressure, system pressure, and density contrast (Figure 4b–d). Yields increased when the pressure was raised from 8 to 12 MPa but started to decrease above 12 MPa. Such a decrease might be a result of reduction in the density contrast, which weakens volume expansion of scCO_2_ caused by ultrasound, adversely affecting exfoliation efficiency. Alternatively, coupling micro‐jet with scCO_2_ was also demonstrated to facilitate exfoliation of graphene with a mechanism similar to ultrasonication.^150^

As an alternative to ultrasound, mechanical shear also can help accelerate exfoliation of layered materials in SCFs.^53^, ^72^, ^151^, ^152^ High rotation speeds of a shear mixer usually mean large shock velocities of SCF molecules parallel to the sheets. As a consequence, these SCF molecules can be wedged into interlayer spaces more readily with large kinetic energy to overcome interlayer vdW attraction. Jet cavitation and collisions that occur during shear mixing may also improve exfoliation in SCFs.^70^ As a proof of this concept, coupling shear force with scCO_2_ appeared to support a claim of high exfoliation yield of graphene of up to 63.2%. About 79% of the flakes were less than five layers, of which monolayer, bilayer, and trilayer represented 27%, 25%, and 14%, respectively. Combining mechanical shear with ultrasound allowed Zhao and co‐workers to increase this claim to a cumulative yield of 80%.^153^

Such rotor–stator devices are popular in industrial fluid processing for formulating emulsions and for small‐scale manufacturing. Other high shear manufacturing methods such as homogenization are used at much higher scales in industrial manufacturing and have yet to be extensively investigated in SCF processing. Homogenization can be envisaged as sequential (cyclical) pressurization to a supercritical state followed by depressurization in a flow that impinges on a mechanical barrier (plate). A very recent paper has addressed an example of this kind of processing.^154^ The homogenization system used is similar to units used in industry to disperse very small batch volumes and imparts shear by driving a graphite‐scNMP multiphase‐fluid through a 3 cm steel capillary (200 µm inner diameter) with cycling fluid flow. Unfortunately, details of this system, other than a cartoon drawing and the capillary dimensions, were not provided. However, similar capillary‐based systems have proven themselves in emulsification for many decades in industry for bench‐top formulation and research.

The results of this small batch study appear promising. Gravimetric yields, obtained by drying and weighing a particular volume of supernatant after 30 min of centrifugation at 3000 rpm, up to 15% were obtained.^154^ These yields, at least, do not depend on visible absorption coefficient. It should be mentioned, however, that yields 800% higher and 33% more concentrated (50 mg mL^−1^) have been reported using sonication in water.^155^ One inconsistency that is difficult to resolve in this study is that TEM of multisheet platelets exhibit the standard and well established intersheet spacing of about 0.35 nm, while the AFM data of exfoliated sheets were interpreted assuming a sheet thickness of 0.8 nm. Such a thickness is consistent with graphene oxide and reduced graphene oxide, but not with pristine graphene. However, the Raman I_D_/I_G_ band ratios of about 0.54 were seen in the starting graphite material and in the exfoliated graphene multisheets. Therefore, pristine graphene appears not to have been available in this study. Further process development using such homogenization and related methods seem to offer promise.

We believe that shear forces derived from fluidic dynamics in SCFs seem a possibly better approach than ultrasound, in light of an absence of sonochemically generated radicals and associated perturbation in nanosheets' quality.^142^ Although such SCF approaches are thought to produce pristine graphene, an absorption coefficient at 660 nm used^70^ to evaluate dispersed graphene of only 16.39 cm^2^ mg^−1^ is only 18% of the absorption coefficient of graphene measured by Nair and co‐workers,^155^ as discussed by Ager and co‐workers.^156^ The experimental results of Nair and co‐workers agree with the fine structure constant‐based value, 91 cm^2^ mg^−1^ in dispersion (2/3 the value for light polarized parallel to a graphene plane), for randomly oriented nanosheets.^156^ This means that these estimates^70^ of yield are fivefold too high.

However, it is reasonable to presume there are many features that affect absorption coefficients of graphite and graphene samples obtained by different methods and from different sources. Some of these variations are manifested in edge and basal plane defects of various types, and their impact on electronic structure, particularly with respect to electronic absorption coefficients (dipole allowed photon absorption probabilities) have yet to be thoroughly evaluated theoretically and experimentally. A more universally accepted means of ascertaining “pristineness” is needed in order to extend our understanding of positive and negative aspects of nanosheet exfoliation processing.

### 3.2 Modification of 2D Materials in SCFs

SCF processing in 2D materials processing has mainly been considered in terms of high‐pressure changes that physically affect interlayer attraction. Additionally, SCFs behave as all solvents behave by modifying distributions of solutes in solution, by causing partitioning among multiple liquid phases, and in modifying chemical equilibria and reaction kinetics. In this section, we focus on reactions of this last type that involve covalent bond formation and scission.

#### 3.2.1 Reduction of GO Using SCFs

Currently, the reduction of GO with hydroxyl, carboxyl, or epoxide groups appears to be the most commonly used route to make graphene‐like materials, that is, reduced graphene oxide (rGO). These modifications can be done through thermal, chemical, electrochemical, or combined thermal–chemical treatments. Thermal methods usually involve high temperatures up to 1000 °C. Chemical reductions can be induced at relatively lower temperatures (less than 100 °C) by using reducing agents. While most strong reductants such as hydrazines and hydrides are highly toxic and explosive, some non‐toxic alternatives such as *l*‐ascorbic acid,^157^ sugar,^158^ protein,^159^ and green tea^160^ require long processing times. It is often difficult to effectively restore C = C π‐conjugation of graphene sheets. Additionally, interfering substances arising from reducing reagents may be strongly physisorbed to rGO, complicating further processing.

SCFs have been demonstrated to be capable of converting GO into rGO, taking advantage of their unique properties (low density and viscosity and diminishing surface tension).^29^, ^161^, ^162^ ScCO_2_ has shown efficiency for such conversion provided the treatment temperature and time are no less than 200 °C and 3 h, respectively.^162^ Indeed the GO films annealed in scCO_2_ at ≥200 °C displayed a significant increase in electrical conductivity, which reached almost 3 S cm^−1^ in the film treated at 300 °C (10 MPa, 5 h) dramatically higher than that in the original GO films (<10^−5^ S cm^−1^).^162^ A mechanistic understanding of deoxygenation of GO in scCO_2_ remains to be established. ScEtOH exhibited a larger reduction activity under similar critical conditions. Electrical conductivity was further improved up to 2.5 S cm^−1^ by using scEtOH (with inherent hydrogen donating ability in the form of molecular hydrogen, hydride, or protons) at 250 °C (10 MPa, 5 h).^162^

**Figure 5:**
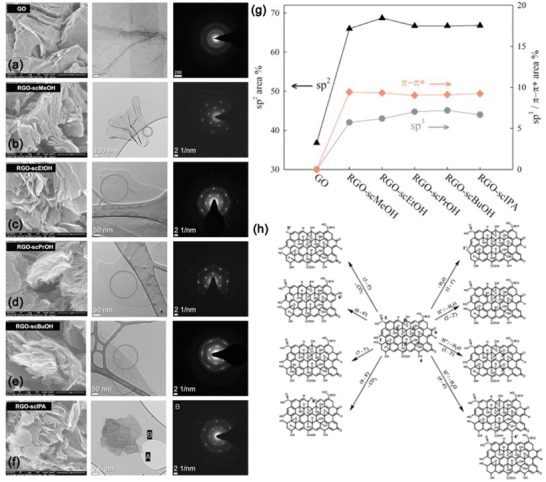
SEM images, HR‐TEM images, and selected area electron diffraction patterns of a) GO, b) RGO‐scMeOH, c) RGO‐scEtOH, d) RGO‐scPrOH, e) RGO‐scBuOH, and f) RGO‐scIPA. g) *Sp* ^2^ and *sp* ^3^ carbon and π–π* shake up of rGO samples. Reproduced with permission.^29^ Copyright 2013, Elsevier Ltd. h) A plausible mechanism for the chemical changes in the GO during subcritical H_2_O (subcH_2_O) and supercritical H_2_O (scH_2_O) treatment. Reproduced with permission.^166^ Copyright 2014, Royal Society of Chemistry.

Of the five different alcohols (methanol [MeOH], ethanol [EtOH], 1‐propanol [PrOH], 2‐propanol [IPA], and 1‐butanol [BuOH]) used at supercritical conditions, scEtOH was found to afford an rGO with the highest carbon‐to‐oxygen ratio of 14.4 and the largest surface area of 203 m^2^ g^−1^ (**Figure** **5**a–g).^29^ The powder conductivity of the resulting rGO in scEtOH was up to 27 500 S m^−1^, 2.5 times higher than that of the rGO treated in scMeOH (10600 S m^−1^),^161^ both of which outperform the electrical conductivity of the rGO sample reduced by hydrazine (≈10000 S m^−1^).^163^ After treatment in scIPA for 1 h, double‐oxidized GO with an O 1s/C 1s ratio of 0.73 and an H/C molar ratio of 7.07 was reduced to an rGO with increased carbon to oxygen ratio (C 1s/O 1s = 12.33) and decreased hydrogen to carbon ratio (H/C = 2.27). This result compares favorably with a thermally annealed sample which showed a lower C 1s/O 1s (10.67) and H/C (0.82).^164^ Such hydrogen‐enriched rGO facilitated Li binding and resulted in high Li^+^ ion uptake, highlighting the importance of this SCF processing. The effective removal of the oxygen functionalities of GO in supercritical alcohols was attributed to a combination of thermal and chemical reduction. The former may involve decarboxylation and hydrodeoxygenation pathways. The latter may be associated with a de‐epoxidation mechanism.^29^

ScH_2_O offers a green alternative to toxic reducing agents.^165^ A higher degree of deoxygenation of GO was attained in subcritical H_2_O and scH_2_O (473–653 K) compared to moderate temperature (373 K) treatment.^166^ Spectroscopic results suggested that the deoxygenation of GO in subcritical H_2_O and scH_2_O could be due to several pathways including hydrogen ion–initiated dehydration by inter‐ or intramolecular reactions, reduction of highly strained epoxide groups, decarboxylation, and generation of conjugated π‐network, as shown in Figure 5h. The addition of glycerol in scH_2_O was found to also enhance GO oxygen removal by up to 59%, resulting from an in situ hydrogen generation.^167^ A very high C/O ratio of 28.2 was obtained by scH_2_O gasification of glycerol to reduce GO sheets (with a C/O ratio of 2.5), outweighing those reported for hydrazine‐based methods.

#### 3.2.2 Nitrogen Doping of Graphene (or GO) via SC Reaction

**Figure 6:**
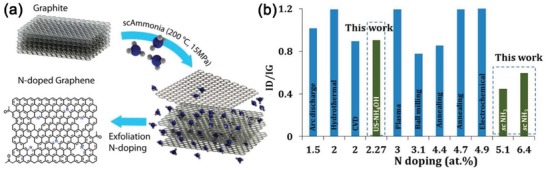
a) Schematic of graphite exfoliation and N doping simultaneously in scNH_3_. b) *I*_D_/*I*_G_ value relative to N doping level of graphene reported by other methods to those of present study. Reproduced with permission.^37^ Copyright 2016, American Chemical Society.

N‐doping of graphene induces polarization in the carbon network due to the higher electronegativity of N (3.04) relative to C. A band gap is opened upon N‐doping, resulting in new properties for catalysis and device applications. SCFs provide unique processing media for chemically doping graphene (or GO) owing to their tunable transport properties. For example, GO was N‐doped by employing N‐containing compounds such as ethylenediamine, melamine, or hexamethylenetetramine^168^ in supercritical ethanol and water solutions (400 °C, 20–25 MPa), with doping levels (at%) of about 4.7, 2.0, and 5.3%, respectively. Using urea,^169^ dimethylglyoxime,^170^ or glycine^171^ as a dopant, similar doping results were obtained in scH_2_O (400 °C). At milder conditions (310 °C), N‐doped few‐layer graphene was obtained in scACN without any addition of other N precursors.^138^ Cyclotrimerization of cyano groups in acetonitrile was inferred to form a 1,3,5‐triazine ring, facilitating the formation of C—N bond primarily at the edges of graphene at high temperature and pressure. The doping content was tuned from approximately 1.6 to 4.6 at% by prolonging reaction time from 2 to 24 h. An even higher N doping level of 6.4 at% was achieved in scNH_3_ (200 °C, 15 MPa) by using graphite as a starting material (**Figure** **6**).^37^ The resulting N configurations were dominated with pyrrolic N (45.9%) followed by graphitic N in the basal plane (32.5%) and also at the edge (10.1%), and pyridinic N (11.5%). However, there was no contribution from oxidized N^+^O^−^ which contrasts the formation of 13.9% pyridinic N^+^O^−^ by doping in ammonia solution under ultrasonication. When using GO instead of graphite in scNH_3_, the doping level was improved to 10.8 at%, comparable to the value obtained by annealing (220 °C) with ammonia gas. Graphene doping types can be changed by adjusting scNH_3_ pressure and by introducing a second phase like CH_4_.^172^

SCFs can promote the doping of many other materials such as TiO_2_.^173^ Nevertheless, relevant studies regarding doping such 2D materials in SCFs are lacking.

#### 3.2.3 Etching of Graphene (or Carbon Flakes) in ScH_2_O

Crystallographic etching of graphene can be achieved based on 1) catalytic (preferably armchair) carbon gasification,^174^, ^175^ and 2) oxidative catalytic etching in scH_2_O.^134^, ^176^

As early as 1955, a variety of metal particles have been investigated for the catalytic graphite–water vapor reaction.^174^, ^175^ Metallic particles move on the surface of graphite during a reaction, leading to the formation of trenches which with the smallest catalyst particles were found to orient mostly in the <1120> directions.^175^ Fe, Co, and Ni were reported as active catalysts for the reaction between 600 and 1000 °C.^175^, ^177^ V and Mo were shown to be weak catalysts under these conditions, while Cu, Zn, Ca, Cr, Mn, and Pb were inactive. Although graphite gasification hardly occurred by Ag NPs in scH_2_O, catalytic carbon oxidation with oxygen was shown to be significantly accelerated by the metallic NPs in scH_2_O.^175^ The etching rate was observed to increase with water density above 0.1 g mL^−1^. Such enhancement of carbon oxidation in scH_2_O was attributed to the efficient removal of adsorbed gaseous products (CO, CO_2_) from the catalyst surface and the formation of hydroxyl (OH) and hydroperoxyl (HO_2_) radicals. This anisotropic etching in combination with exfoliation in scH_2_O allows the scalable production of zigzag‐edge‐rich graphene nanosheets for electrochemical devices.^176^

ScH_2_O treatment of anthracite coal (400 °C, 25 MPa, 120 min) was observed to result in single‐layer graphene oxide quantum dots (GQDs).^178^ ScH_2_O cutting down of large anthracite flakes started as early as 10 min. The depolymerization of coal was hypothesized to result from cleavage of ether and carbon−carbon (C—C) bonds induced by the nonpolar scH_2_O. In contrast to commonly used oxidizing agents, the scH_2_O oxidation was selective and did not bring about severe aromatic ring degradation. Exfoliation occurred at the same time, which led to a significant reduction in the number of layers for the GQDs upon extension of treatment.

#### 3.2.4 SCF‐Induced Phase Engineering

Phase transformations of layered materials have been realized in SCFs. It has been reported that scDMF boosts oxygen release in layered MnO_2_, enabling a phase change from MnO_2_ to Mn_3_O_4_ in short periods (400 °C, <10 min)^66^ and further to MnO after extended processing times (>10 min).^179^ In scDMF, a chemical phase change occurs, and morphology (nanocrystal habit) transformations occur in kinetic competition with one another. MnO_2_ to Mn_3_O_4_ to MnO changes show that a reduction occurs from Mn^+4^ to mixed Mn^+3^ and Mn^+2^ to Mn^+2^, respectively.^180^ Accompanying oxidation processes were not identified. Starting nanosheet layers are transformed into nanocrystals of various shapes. This diversity is driven by phase change and by Ostwald ripening. Ripening is promoted by solvation of chemical units (ions, complexes, molecules) in scDMF (or other solvents in other 2D material systems).

**Figure 7:**
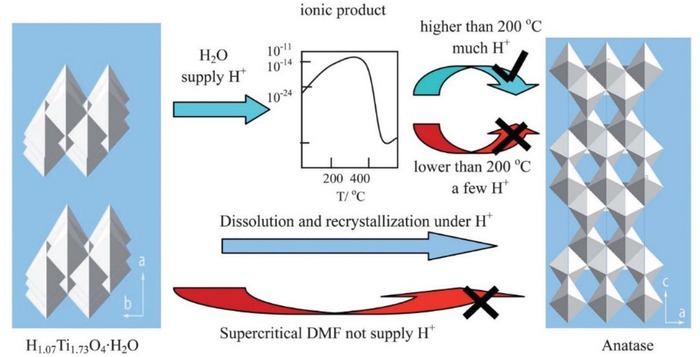
Schematic of the H_1.07_Ti_1.73_O_4_·H_2_O phase transition process under scH_2_O and scDMF. Reproduced with permission.^62^ Copyright 2013, Royal Society of Chemistry.

Compared to scDMF which exfoliates titanate without phase change, scH_2_O was found to trigger a phase transformation of H_1.07_Ti_1.73_O_4_·H_2_O into anatase above 200 °C.^62^ This was presumably due to dissolution and recrystallization (Ostwald ripening) caused by H^+^ that was supplied by scH_2_O, as illustrated in **Figure** **7**.

Protonation often increases solubility in polar solvents, and can then expected to promote Ostwald ripening.

SCFs also offer external stimuli to facilitate the formation of 2D lateral heterostructures for enhanced photocatalysis.^35^, ^65^, ^67^, ^78^, ^181^, ^182^, ^183^, ^184^ For example, scCO_2_‐ethanol‐water mixed solvents provided benefits for the production of 2H‐WS_2_ nanosheets with abundant edges.^35^ Those exfoliated monolayers were readily oxidized by the air remaining in the system to form WO_3_, yielding 2D WS_2_/WO_3_·H_2_O lateral heterostructures. High‐resolution transmission electron microscopy (HRTEM) revealed irregular holes or vacancies on the basal planes of WO_3_·H_2_O nanosheets.^182^ The presence of oxygen deficiency on the surface was further confirmed by X‐ray photoluminescence spectroscopy. The formed H_3_O^+^ originating from the reaction of water and CO_2_ may initiate defect generation via an electrophilic attack.

**Figure 8:**
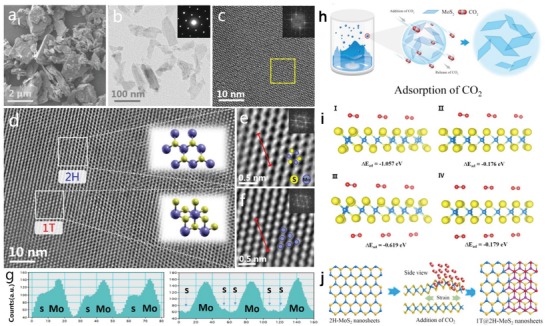
a) SEM image of bulk MoS_2_ powder. b) TEM and c) HRTEM images of MoS_2_ nanosheets. Inset: FFT of the region enclosed by the yellow square. d) HRTEM image of MoS_2_ heterostructure. Inset: Schematic structures of the unit cells of 1T and 2H MoS_2_. e,f) Filtered images of the regions enclosed by white squares shown in (d). g) Intensity distribution along the red dashed line in (e,f). h) Schematic of the 1T@2H‐MoS_2_ heterostructure formation mechanism. i) Adsorption of CO_2_ on a single‐layer MoS_2_ (2*2) surface: CO_2_ is adsorbed on one side of I) 1T‐MoS_2_ and II) 2H‐MoS_2_, and on two sides of III) 1T‐MoS_2_ and IV) 2H‐MoS_2_. j) Schematic of the top and side views of MoS_2_ with strained S vacancies on the basal plane by using scCO_2_. Reproduced with permission.^181^ Copyright 2016, American Chemical Society.

ScCO_2_ was also found to induce a phase transition of semiconducting (trigonal prismatic) 2H‐MoS_2_ to metallic (octahedral) 1T‐MoS_2_ (**Figure** **8**).^181^, ^184^, ^185^ No defects or deformation of the lattice structure of MoS_2_ were observed during the process (Figure 8c–f). The adsorption energy of CO_2_ on the 1T‐MoS_2_ (−0.62 eV) was calculated to be more than three times stronger than that on the 2H‐MoS_2_ (−0.18 eV) (Figure 8i). This difference in CO_2_ adsorption strength was assumed to cause such phase conversion. The obtained 1T MoS_2_ nanosheets were speculated to be stabilized by the adsorbed CO_2_. However, how to control the yield of 1T‐MoS_2_ has not been addressed. A similar concept has been demonstrated to be effective in the construction of amorphous MoO_3_ nanosheets with the help of scCO_2_.^65^, ^186^ The coexistence of metastable *h*‐MoO_3_ and stable α‐MoO_3_ resulting from the oxidation of exfoliated MoS_2_ likely played a role.^65^ Such conjecture was based on the finding that starting from pure MoO_3_ nanosheets only yielded an orthorhombic crystalline structure rather than an amorphous structure after scCO_2_ treatment. The stronger adsorption of CO_2_ on an amorphous surface than on crystal surface was supposed to favor stabilization of the amorphous MoO_3_.^187^, ^188^ It may also induce diffusive atomic disordering of MoO_3_ nanosheets.

### 3.3 Molecular Modeling of SCF‐Assisted Exfoliation

Wu and Yang provided some significant molecular dynamics simulation results where scCO_2_ was examined as a solvent for two model graphene sheets at a critical density, ρ_c_ (0.468 g cm^−3^), and at 2ρ_c_, at three temperatures, 45, 90, and 135 °C.^129^ These thermal variations were inconsequential, but density (pressure) was found to have a significant effect, with intersheet repulsion increasing with density. They quantitatively examined free energy solvent effects through a calculated potential of mean force (PMF) by partitioning it into a sheet–sheet component and an scCO_2_‐induced component. This second component accounts for density effect and arises from the formation of molecular layers of scCO_2_ on the sheet surfaces.^129^ These authors found that as density increased additional solvent layers preferentially formed on the sheets, and this sheet solvation accounts for stabilizing maxima in the intersheet repulsion at separations greater than 6.5 Å. They also concluded that intersheet solvent intercalation is a critical step in exfoliation. This layer‐solvation effect was discussed by Israelachvili and co‐workers many years ago in terms of a “solvation force.” These workers explored specific solvation effects between crossed cylinders in initial versions of a surface forces apparatus. Intersheet repulsion forces were found to exhibit periodic maxima corresponding to solvent layer thicknesses.^189^, ^190^ They also found a similar periodic variation in repulsion when they examined C_60_ fullerene multilayers on clay surfaces.^191^ We can thereby understand graphene stability in scCO_2_ in terms of fundamental colloid science, Israelachvili's multilayer solvation force. Similar MD (molecular dynamics) studies of other supercritical solvents will be very useful, particularly if solvents exhibiting overlapping π binding to graphene's *sp* ^2^ surfaces can be modeled.

An initial examination of such π binding solvation under near‐critical and supercritical conditions has been described by Xu and co‐workers^192^ in steered molecular dynamics (SMD)^31^ simulations as a function of sheet separation for a polyethyleneglycol‐pyrene (Py‐PEG) polymeric conjugate. These SMD simulations included subcritical and supercritical conditions; they included mixtures of CO_2_, PX (*p*‐xylene), and Py‐PEG, as well as examinations of CO_2_ and PX alone. The scCO_2_‐alone simulations corroborated some of Wu and Yang's finding^129^ that initial separations less than 6.5 Å evolved to an exclusion of CO_2_ and de facto association of graphene sheets with an interlayer separation of about 4 Å (because of an scCO_2_‐induced intersheet repulsive barrier at about 6.5 Å). Initial separations of 10 Å also evolved to an average separation of about 4 Å (first to about 6.5 Å and then to 4 Å) at a density of 0.912 g cm^−3^, above ρ_c_ and only slightly lower (2.6%) than 2ρ_c_ (0.936 g cm^−3^). This discrepancy was not discussed by Xu and co‐workers but may be a consequence of their nanosheet constraints. A conclusion that scCO_2_ cannot stabilize graphene nanosheets appears contrary to many experimental observations. The detailed discussion of Wu and Yang describes a step‐wise wedge‐opening effect followed by surface solvation by scCO_2_,^129^ We emphasize that this repulsive barrier at about 6.5 Å was also observed at only 0.468 g cm^−3^ (ρ_c_). The only PMTs provided by Xu and co‐workers^192^ were expressed in terms of a “sliding distance.” It is unclear whether this coordinate corresponds to nanosheet separation (which happens along the *y*‐coordinate, normal to nanosheets) or to sliding of one sheet past the other at an imposed velocity of 2.5 Å ns^−1^ in the *x*‐coordinate direction. Values of PMF ranged from 0 to about 25.6 kJ mol^−1^ nm^−2^,^192^ which is only a 25% potential energy change calculated between the nanosheet aggregation state energy minimum at about 4 Å separation to a primary maximum at 7.5 Å separation, and an energy difference of about 100 kJ mol^−1^ nm^‐2^ (23.9 kcal mol^−1^ nm^‐2^).^129^ Whatever these calculated^192^ PMFs are, the “sliding distance” cannot be nanosheet separation along a *y*‐coordinate because the energies of these PMFs go to zero as “sliding distance” goes to zero. Additionally, in neither case is a ceiling to a steadily increasing barrier shown, since the maximum sliding distance illustrated is 6 Å.

Xu and co‐workers also examined mixtures of CO_2_ and PX and CO_2_, PX, and Py‐PEG. They found that PX alone was not effective in initiating exfoliation with initial layers spaced 6 Å apart, but at an initial separation of 10 Å, the sheets evolved to a 7.4 Å separation, indicating that at least a monolayer of PX separates the respective sheets. Mixed CO_2_/PX solutions were useful when the [CO_2_]/[PX] ratio was higher than 2.5. At such higher concentration ratios, the nanosheet spacing evolved to about 7.5 Å when starting from 6 Å, and to 10.5 Å when starting from a 10 Å interlayer spacing. Unfortunately, PMF was not calculated for these solvent compositions. A qualitatively significant advance was in analyzing stabilization by Py‐PEG, when mixed CO_2_/PX solvents initiated exfoliation. These simulations showed that the pyrene head groups provided π–π binding that survived venting of CO_2_.^192^ This same kind of binding can be obtained for many pyrene derivatives of varying charges.^31^ Essentially, any conjugated ring system is a suitable candidate for π–π adsorption or anchoring to graphene surfaces. Suitably strong binding moieties for 2D material surfaces somewhat dissimilar from graphene must be found empirically.

### 3.4 Process Opportunities

It appears that simple RESS processing can induce exfoliation and that cyclical repeating of such processing has an additive effect resulting in progressively increased exfoliation extents. SCFs with mild critical properties that are gases under ambient conditions offer important processing advantages. Using co‐solvents with some of these “mild” SCFs provides some apparent advantages, including formation of complex fluids (using co‐solvents immiscible with SCFs that form emulsions, polymer solutions). Also, coupling shear‐generating processing methods with SCF processing appears to be a very useful area for process development.

Further work is needed in understanding and characterizing covalent bond formation accompanying extant solvent processing of 2D materials, and this need is also a development opportunity for many SCF processes applied to such materials. Shear‐induced bond breaking, sonolysis‐induced solvent radical formation, reactions of radicals with 2D sheets in SCF and SCF‐cosolvent mixtures, and redox reactions in SCF are examples of chemistries that would benefit from further understanding in connection with 2D material processing in SCFs.

More physical and chemical modeling and simulation on different length scales are needed. CFD helps us understand microscopic fluid dynamics and how macroscopic waves and shear‐fields can be generated and transmitted in multiphase suspensions.^69^, ^71^ Molecular dynamics simulations have also advanced so that nanoscopic processes can be examined and correlated with macroscopic supercritical parameters.^49^, ^129^, ^192^ One of these studies has provided a clear picture of how SCF molecules produce free energy potentials that promote exfoliation.^129^

## 4 Formation and Applications of 2D Materials Processed by SCFs

Applications of nanosheets prepared or processed with SCF assistance are mirroring those of nanosheets created by other top‐down and bottom‐up approaches. Catalysis and energy account for a large majority of these applications and analytical applications such as imaging and sensors represent another primary focus. Sasikala and co‐workers^17^ have recently discussed supercritical fluid applications of graphene in conductive films^128^, ^133^ and field effect transistors, batteries, capacitors, solar cells, fuel cells, biological and environmental applications, sensors, catalysis (an Mo_2_C/rGO material obtaining hydrocarbons from glycerides),^193^ and microwave absorption devices. A novel heterostructured material composed of graphene nanosheets encapsulated with MoS_2_ nanosheets is a noteworthy advance in superhydrophobicity.^39^ Sathish and co‐workers noted that coatings of these encapsulated sheets exhibited superhydrophobicity (water contact angle of 166°). The component nanosheets BN and MoS_2_ separately yielded contact angles of 125°. This synergism is most likely due to a “lotus” effect that often accompanies a very rough surface topography,^194^ a property that often accompanies 3D heterostructures. However, a key property of these mixed sheet materials is obtaining heterojunction contacts of basal planes.

In many of these applications (**Table** **4**), 2D materials are major components of electrodes, and their electrical conductivity is a necessary property. We also discuss some particular types of composite materials, wherein 2D nanosheets provide flame retardancy. Lubricants compose a type of multiphase materials, sometimes multiphase fluids, and we discuss those containing nanosheets.

**Table 4:** Summary of SCF‐processed 2D materials reported for various applications

2D materials and hybrid	Precursor	Experimental condition	(Application) Performance	Ref.
Pt (or Ru)–rGO	C_10_H_18_Pt, C_3_0H_50_O_4_Ru, rGO	ScCO_2_, 13 MPa, 60 °C, 24 h	(Limonene hydrogenation) Conversion: 57% (Pt–rGO) and 54% (Ru–rGO) *p*‐Menthene selectivity: 85% (Pt–rGO) and 87% (Ru–rGO) Yield: 48% (Pt–rGO) and 47% (Ru–rGO) TOF: 43 × 10^−3^ h^−1^ (reaction time: 30 min)	105
Pt–rGO	Pt(C_5_H_7_O_2_)_2_, rGO	ScCO_2_, 12 MPa, 200 °C, 3 h	(Methanol electro‐oxidation) Onset potential: 0.17 V (vs Ag/AgCl) ECSA: 41.5 m^2^ g^−1^ *I*_f_ (forwarded peak current density): 44.8 mA cm^−2^ Mass activity: 550.4 mA mg_Pt_^−1^	195
Pt–rGO	C_10_H_18_Pt, rGO	ScCO_2_, 24.5 MPa, 70 °C, 6 h	(Methanol electro‐oxidation) ECSA: 44.3 m^2^ g^−1^ Onset potential: 0.13 V (vs Ag/AgCl) *I*_f_: 22.1 mA cm^−2^ *I*_f_/*I*_b_: 1.43 Mass activity: 202.2 mA mg_Pt_^−1^	196
Pd (or Fe, Ni, Pd, and Au)–rGO	Pd(C_5_HF_6_O_2_)_2_, Fe(C_5_H_7_O_2_)_3_, Ni(C_5_H_7_O_2_)_2_, AuCl_3_, GO	ScCO_2_–methanol, 10 MPa, 50 °C, 2 h, dimethyl amineborane as reductant	(Dehydrogenation of LiAlH_4_) 4.5 wt% hydrogen desorption time: 0.3 h (2.5 wt% of Fe–rGO) and 6 min (10 wt% of Fe–rGO)	100
Pd–graphene	Pd(C_5_HF_6_O_2_)_2_, graphene	ScCO_2_–NMP/methanol, 18 MPa, 50 °C, 5 h, dimethyl amine borane as reductant	(Formic acid electro‐oxidation) ECSA: 103.8 m^2^ g^−1^ *I*_f_: 84.7 mA cm^−2^ Mass activity: 1045.7 mA mg_Pd_^−1^ (Methanol electro‐oxidation) ECSA: 115.0 m^2^ g^−1^ *I*_f_: 66.9 mA cm^−2^ Mass activity: 823.4 mA mg_Pd_^−1^	102
Pd–rGO	Pd(C_5_HF_6_O_2_)_2_, rGO	ScCO_2_–H_2_, 4000 psi, 45 °C, 24 h, then scCO_2_, 4000 psi, 45 °C, 24 h	(Suzuki reaction) Yield: 98.5% (100 °C, 5 min, 5.1 wt% Pd–rGO) Recyclability: 79.1% (yield) after 10 cycles	197
Ag–GO	AgNO_3_, GO	ScCO_2_–ethanol, 12 MPa, 65 °C, 3 h, glucose as reductant	(Photocatalytic dye degradation) Rhodamine 123 dye degradation efficiency: 82% Acetaldehyde degradation efficiency: 93% (after 60 min of visible light illumination)	198
PtRu–rGO	Pt(C_5_H_7_O_2_)_2_, Ru(O_2_C_5_H_7_)_3_, rGO	ScCO_2_–H_2_–methanol, 12 MPa, 200 °C, 1.5 h, then 16 MPa, 30 min	(Methanol electro‐oxidation) Onset potential: 0.12 V (vs Ag/AgCl) *I*_f_/*I*_r_: 6.75, 3.45, and 2.64 (first, fifth, and tenth cycles)	199
PtFe–graphene	Pt(CF_3_COCHCOCF_3_)_2_, Fe(C_5_H_7_O_2_)_3_, honeycomb‐structured graphene (HSG)	ScCO_2_–tetrahydrofuran (THF), 30 MPa, 333 K, 2 h, then 15 MPa, 353 K, 30 min, borane‐THF as reductant	(Oxygen reduction reaction, ORR) ECSA: 110 m^2^ g_Pt_^−1^ (Pt_40_Fe_60_/HSG) Onset potential: 0.989 V (vs RHE) Half‐wave potential: 0.943 V (vs RHE) Mass activity: 1.70 A g_Pt_^−1^ (at 0.9 V) Specific activity: 1.55 mA cm^−2^ (at 0.9 V) ECSA loss: 9.0% after 10 000 cycles	107
PtFeCo–graphene	Pt(CF_3_COCHCOCF_3_)_2_, Co(C_5_HF_6_O_2_)_2·_xH_2_O, Fe(C_5_H_7_O_2_)_3_, graphene cellular monolith	ScCO_2_–ethanol, 25 MPa, 323 K, 2 h, then 333 K, 1 h, borane‐THF as reductant	(ORR) Half‐wave potential: 0.916 V (vs RHE) Mass activity: 1.28 A mg_Pt_^−1^ Specific activity: 1.80 mA cm^−2^	200
ZnO–rGO	Zn(NO_3_)_2_·6H_2_O, GO	ScCO_2_–ethanol, 9 MPa, 300 °C, 6 h	(Photocatalytic hydrogen production) Activity: 28.9 µmol g^−1^ (2 h)	101
Co_3_O_4_–GO	Co(NO_3_)_2_·6H_2_O, GO	ScCO_2_–ethanol, 9 MPa, 150 °C, 24 h	(Ammonium perchlorate decomposition) Decomposition temperature: 297 °C Exothermic heat: 1591 J g^−1^	96
Fe–TiO_2_–rGO	(C_12_H_28_O_4_)Ti, FeCl_3_, rGO	ScCO_2_–IPA/acetic acid, 5000 psig, 60 °C, 24 h	(E2 photodegradation) Half‐life of E2: 41 min (1 sun intensity)	201
TiO_2_–graphene	(C_12_H_28_O_4_)Ti, graphene	ScCO_2_–ethanol/H_2_O, 23 MPa, 350 °C, 8 h	(Methyl orange dye photodegradation) Efficiency: 100% (180 min)	202
Mo_2_C–rGO	C_10_H_16_MoO_6_, GO	SC 2‐methyl‐1‐propanol, 400 °C, 30 min	(Oleic acid deoxygenation) HC selectivity: ≥90% HC yield: ≥85%	193
CdS–rGO	C_4_H_6_CdO_4_·2H_2_O, Na_2_S_2_O_3_·5H_2_O, rGO, glutathione	ScCO_2_–H_2_O, 12.5 MPa, 75 °C, 90 min	(Photoelectrochemical water splitting) Photocurrent densities: at least three times higher than pristine CdS NPs	103
N–doped graphene (NG)	Graphite	ScNH_3_, 15 MPa, 200 °C, 1–2 h	(ORR) Reduction peak: −0.30 V (vs Ag/AgCl) Electron transfer number: 3.62	37
1T@2H MoS_2_	MoS_2_ nanosheets	ScCO_2_–ethanol/H_2_O, 353.2 K, 6 h	(Photoelectrocatalytic water splitting) Photocurrent densities: −1400 µA cm^−2^ (at −0.6 V) Photocurrent response: 1.2 × 10^−5^ A cm^−2^	181
2H–/1T′–MoS_2_–graphene	Graphene, 2H‐MoS_2_ nanosheets	ScCO_2_–ethanol/H_2_O, 20 MPa, 433.2 K, 6 h	(Photocatalytic hydrogen evolution) Photocurrent response: 51.0 µA cm^−2^	184
2H@1T–MoS_2_–graphene	Graphite, MoS_2_ powder, PVP	ScCO_2_–ethanol/H_2_O, 16 MPa, 40 °C, 3 h	(Photoelectrocatalytic hydrogen evolution) Photocurrent response: 5.5 × 10^−5^ A cm^−2^ Activity: 19.82 mmol g^−1^ h^−1^	78
WS_2_–WO_3_·H_2_O/1T–2H MoS_2_	WS_2_ powder, MoS_2_ powder	ScCO_2_–ethanol/H_2_O, 16 MPa, 313.2 K, 3 h	(Photoelectrocatalytic hydrogen evolution) Photocurrent response: 10.6 × 10^−5^ A cm^−2^	67
WS_2_/WO_3_·H_2_O	WS_2_ powder	ScCO_2_–ethanol/H_2_O, 16 MPa, 313.2 K, 3 h	(Methyl orange photodegradation) Degradation: 90% (40 min, UV irradiation or 240 min, visible‐light irradiation) Photocurrent response: 6 × 10^−5^ A cm^−2^	35
rGO	GO	ScMeOH, 30 MPa, 400 °C, 15 min–2 h	(Anode in LIBs) Discharge capacity: 652 mA h g^−1^ at 50 mA g^−1^ after 40 cycles	161
Hydrogen‐enriched rGO	GO	ScIPA, ultrasonication: 1 h, then 400 °C, 1 h	(Anode in LIBs) Reversible capacity: 1331 mAh g^−1^ at 50 mAg^−1^ after 100 cycles Rate‐performance: 328 mAh g^−1^ at 5 A g^−1^ Cycling stability: 1000 cycles at 10 A g^−1^	164
Si nanowire–graphene	Diphenylsilane, GO	SC hexane, 360 °C, 1 h	(Anode in LIBs) Reversible capacity: 1400 mAh g^−1^ for the 30th cycle at 420 mA g^−1^	203
Hydrogen‐enriched porous carbon nanosheets	Natural graphite, KMnO_4_, H_2_O_2_, H_2_SO_4_	scIPA, ultrasonication: 1 h, then 400 °C, 1 h	(Anode in sodium ion batteries) Reversible capacity: 300 mAh g^−1^ at 50 mA g^−1^ Cycling stability: 2000 cycles at 1–5 A g^−1^	204
1‐Pyrene sulfonic acid sodium salt modified graphene	Graphite powder, 1‐pyrene sulfonic acid sodium salt	ScEtOH/H_2_O, 450 °C, 2 h	(Anode in LIBs) Saturated reversible capacity: 301.25 mAh g^−1^ at 0.01 mA g^−1^	74
SnO_2_–rGO	Tin(II) acetate, GO	ScMeOH, 400 °C, 30 min	(Anode in LIBs) Reversible discharge capacity: 776 mAh g^−1^ after 70 cycles at 0.1 A g^−1^ Rate‐performance: 147 mAh g^−1^ at 5 A g^−1^ Cycling performance: 531 mAh g^−1^ after 1000 cycles at 1 A g^−1^	205
CoCO_3_–rGO	Co(CH_3_COO)_2_, rGO	ScCO_2_–ethanol, 50 °C, 10 MPa, 2 h	(Anode in LIBs) Reversible discharge capacity: 745 mAh g^−1^ at 6 A g^−1^ Coulombic efficiency: >95% after 2 cycles (Anode in NIBs) Reversible discharge capacity: 370 mAh g^−1^ at 50 mA g^−1^	206
LiFePO_4_–graphene	Graphite powder, LiFePO_4_ particles	ScCO_2_, 40 °C, 8 MPa, 30 min, CF: 1500 rpm, 60 min	(Cathode in LIBs) Discharge capacity: 160 mAh g^−1^ at 0.1 C over 15 cycles	32
LiFePO_4_/C, LiMnPO_4_/C, LiCoPO_4_/C, LiNiPO_4_/C	(NH_4_)_3_PO_4_·3 3H_2_O, FeSO_4_·7H_2_O, MnSO_4_·H_2_O, CoSO_4_·7H_2_O, NiSO_4_·6H_2_O, CH_3_COOLi·H_2_O	ScEtOH–PVP, 10 MPa, 400 °C, 2 h	(Cathode in LIBs) Discharge capacity: 70 mAh g^−1^ at 80 C (LiFePO_4_/C); 40 mAh g^−1^ at 30 C (LiMnPO_4_/C); 53 mAh g^−1^ at 20 C (LiCoPO_4_/C)	60
V_2_O_5_	V_2_O_5_ powder	ScEtOH, 250 °C, 12 h	(Cathode in LIBs) Initial capacity: 90 mA h g^−1^ at 15 C 100% capacity retention after 200 cycles	63
Li_2_MnSiO_4_ (M = Fe, Mn)−multiwalled carbon nanotubes (MWNTs)	FeCl_2_·4H_2_O, Si(OC_2_H_5_)_4_, LiOH·H_2_O, ascorbic acid, MWNTs	350–420 °C, 38 MPa, 4–10 min, 300 °C, 3 h under Ar	(Cathode in LIBs) Discharge capacity: ≈340 mAh g^−1^ at 45 ± 5°C Cycle ability: 20 cycles	207
MoS_2_, MoSe_2_	Bulk MoS_2_ and MoSe_2_	ScDMF, ultrasonication (160 W): 5 min, then 400 °C, 1 h, CF: 2000 rpm, 30 min and 30 000 rpm, 1 h	(Cathode in Mg−Li ion batteries) Discharge capacities: 81 mAh g^−1^ (MoS_2_) and 55 mAh g^−1^ (MoSe_2_) at 20 mA g^−1^	56
Graphene aerogel	GO	ScCO_2_ drying followed by H_2_ reduction at 1100 °C for 1 h	(Supercapacitor) Specific capacitances: 153 (ionic liquid ([EMIM]TF_2_N)) and 90 F g^−1^ (1 m MeEt_3_NBF_4_/PC) at 100 mA g^−1^ Energy density: 21.4 Wh kg^−1^ at 100 mA g^−1^ ([EMIM]TF_2_N)	208
Nitrogen‐doped graphene oxide (NGO)	GO, ethylenediamine or melamine or hexamethylenetetramine	ScH_2_O/ethanol, ultrasonication: 30 min, then 20–25 MPa, 400 °C, 30 min	(Supercapacitor) Specific capacitances: 280 F g^−1^ in aqueous 1 M H_2_SO_4_ (0.9 V) and 104 F g^−1^ in ionic liquid EMITFSA (3.6 V) Energy densities: 8 (1 M H_2_SO_4_) and 40 W h kg^−1^ (EMI‐TFSA)	168
Nitric acid treated–NGO	GO, urea, HNO_3_	ScH_2_O, ultrasonication: 30 min, 400 °C, 20–25 MPa, 1 h	(Supercapacitor) Specific capacitance: 261 F g^−1^ at 0.5 A g^−1^	169
NGO	GO, dimethylglyoxime	ScH_2_O, 400 °C, 2 h	(Supercapacitor) Maximum specific capacitance: of 286 F g^−1^ at 0.5 A g^−1^ Cycling results: 98% specific capacity retention, 100% coulombic efficiency over 1000 cycles at 5 A g^−1^	170
NGO	GO, glycine	ScH_2_O, 400 °C, 1 h	(Supercapacitor) Specific capacitance: 270 F g^−1^ at 0.5 A g^−1^ Capacitance retention: 90% over 10 000 cycles at 10 A g^−1^ Symmetric supercapacitor cell energy density: 4.1 and 36 Wh kg^−1^ in aqueous and ionic liquid electrolytes, respectively	171
ZnO–rGO	Zn(NO_3_)_2_·6H_2_O, GO	ScCO_2_–ethanol, 9 MPa, 300 °C, 6 h	(Supercapacitor) Specific capacitance: 303 F g^−1^ at 10 A g^−1^ Cycling stability: 1000 cycles at 10 A g^−1^	99
MnO_2_–graphene	KMnO_4_, graphene	ScCO_2_–ethanol, 12 MPa, 50 °C, 30 min	(Supercapacitor with ionic liquid EMI‐TFSI additive) Specific capacitances: 230 (at 50 mV s^−1^) and 207 F g^−1^ (at 500 mV s^−1^) Cycling stability: 98% of capacitance retained after 10 000 cycles at 50 mV s^−1^	209
Polypyrrole‐coated GO–carbon nanofiber films (GC–SC/PPy)	GO, carbon nanofiber, pyrrole	ScCO_2_, 16 MPa, 40 °C, 3 h, then pyrrole polymerization in the presence of HCl and ammonium persulfate (APS)	(Supercapacitor) Specific capacitances: 144.6 F g^−1^ (GC–SC/PPy–50) at 10 mV s^−1^ Cycling stability: 89% of initial capacitance after 5000 cycles at 1 A g^−1^	210
GO–polyaniline	GO, aniline	ScCO_2_–ethanol/H_2_O, ultrasonication: 30 min, then 12 MPa, 40 °C, 3 h in the presence of HCl and APS	(Supercapacitor) Specific capacitance: 425 F g^−1^ at 0.2 A g^−1^ Cycling stability: 83% of initial capacitance after 500 cycles at 1 A g^−1^	211
Graphene	Graphite crystals	ScNMP/scEtOH/scDMF, ultrasonication (160 W): 10 min, then 38–40 MPa, 300–400 °C, 3 min	(Electronic devices) Sheet resistance: 2–6 kΩ	133
Graphene	Graphite powder	ScCO_2_, 40 °C, 12 MPa under ultrasonication (120 W), 60 min	(Conductive films) Electrical conductivity: 6.6 × 10^2^ S m^−1^ (37 nm film thick) and 2.8 × 10^7^ S m^−1^ (>300 nm film thick)	128
NG	Expanded graphite, acetonitrile	ScACN, 310 °C, 2–24 h	(Electronic devices) Sheet resistance: ≈300 Ω/⬜	138
MoS_2_	Bulk MoS_2_	ScDMF, ultrasonication: 5 min, then 400 °C, 1 h	(Electronic devices) Charge carrier mobility: ≈1530 cm^2^ V^−1^ s^−1^	55
Eu^3+^ doped CaTiO_3_	Ca(NO_3_)_2_·4H_2_O, Eu(NO_3_)_3_·6H_2_O, Ti(OC_4_H_9_)_4_	ScN_2_, 1 MPa, 250 °C, 4 h, then 265 °C, 1.5 h, then calcination in air, 800 °C, 4 h	(Luminescence imaging) Red emission at 616 nm (excited at 466 nm)	212
MoS_2_	MoS_2_ powder	ScCO_2_–ethanol/H_2_O, ultrasonication: 2 h, then 16 MPa, 313.2 K, 3 h, and ultrasonication: 3 h, CF: 3000 rpm, 15 min	(Luminescence imaging) Emission peaks at 395 and 572 nm (excited in the range 300–550 nm)	79
MoS_2_	MoS_2_ powder	ScDMF, ultrasonication: 5 min, then 400 °C, 30 min, CF: 2000 rpm, 20 min	(Luminescence imaging) Blue emission peak at 420 nm (excited at 360 nm)	143
Pd–rGO	(C_10_H_2_F_12_O_4_)Pd, rGO	ScCO_2_–methanol, 10 MPa, 50 °C, 2 h, dimethylamine borane as a reductant	(Sensors) Detection of 1 mm ascorbic acid (AA), 2 µm dopamine (DA), and 50 µm uric acid	97
Ionic liquid modified Pd–rGO	(C_10_H_2_F_12_O_4_)Pd, rGO, butylmethylpyrrolidinium–bis(trifluoromethanesulfonyl)imide(BMP–TFSI), butylmethylpyrrolidinium–dicyanamide(BMP–DCA)	ScCO_2_–methanol, 10 MPa, 50 °C, 2 h	(Sensors) Selective detection of glucose or AA from their mixture. BMP–TFSI is selective for glucose detection; BMP–DCA is selective for AA detection	104
Ionic liquid modified Pd–rGO	(C_10_H_2_F_12_O_4_)Pd, GO, 1‐butyl‐3‐methylimidazolium hexafluorophosphate (BMI–PF6)	ScCO_2_–methanol, 10 MPa, 50 °C, 2 h	(Sensors) DA detection selectivity: 3.28 µA µm ^−1^ DA detection limit: 0.12 µm	213
Au–rGO	HAuCl_4_·3H_2_O, rGO	ScCO_2_–methanol, 10 MPa, 50 °C, 1 h	(Sensors) Glucose detection sensitivities: 16.3 µAm m ^−1^ cm^−2^ and 97.8 µAm m ^−1^ cm^−2^ (with ionic liquid additive) Detection limits: 0.183 µm and 0.062 µm (with ionic liquid additive)	214
RGO–Prussian blue aerogels	GO, K_3_[Fe(CN)_6_], FeCl_3_, *l*‐ascorbic acid	ScCO_2_ drying	(Sensors) H_2_O_2_ detection limit: 5 nm A wide linear range (0.005–4 mm)	115
Al_2_O_3_–rGO	Al(NO_3_)_3_·9H_2_O, GO	ScCO_2_, 20 MPa, 300 °C, 30 min	(Sensors) Chemical sensing ethanol concentration: 1.5 µg mL^−1^ at 200 °C Response time: about 10 s Recovery time: <100 s	215
Ag–rGO	AgNO_3_, GO	ScCO_2_–ethanol, 15 MPa, 80 °C, 1 h in the presence of glucose and NH_3_·H_2_O	(Lubricants) Friction coefficient: 30.4% Wear scar diameter: 27.4%	84

### 4.1 Catalysis

Catalysis with 2D nanosheets has gained increasing research interest because of their remarkable properties and flexibility in surface and structure modification by diverse methods.^3^, ^4^ By using SCFs, the surfaces of graphene, rGO, or GO have been decorated with a variety of metal (such as Pt,^105^, ^195^ Ru,^105^ Pd,^97^, ^100^, ^102^, ^197^ Ag,^84^, ^198^, ^216^, ^217^ Au,^100^ Fe,^100^ Ni,^100^, ^105^ PtRu,^199^ PtFe,^107^ and PtFeCo^200^), metal oxide (such as ZnO,^99^, ^101^, ^218^ Co_3_O_4_,^96^ TiO_2_,^201^, ^202^, ^219^ MoO_2_,^193^ MnO_2_,^209^ Al_2_O_3_,^215^ and SnO_2_,^205^), and metal sulfide (such as CdS^103^) NPs with tunable sizes, loadings, and compositions. These supported catalysts hold promise for many different catalytic processes. For example, rGO supported Pt NPs of 3.5–5.4 nm and Ru NPs of 2.5–4.4 nm were demonstrated to be active for limonene hydrogenation in scCO_2_ with a selectivity of ≈90% for *p*‐menthene.^105^ Both catalysts exhibited higher conversions (≈78% at 60 min) than commercial Ru/C (42%) and rGO (38%). The Ru/rGO catalyst was reused four times with only a minor loss of performance. Such good activity and selectivity may be related to the 2D open structure of the rGO support, which favors adsorption of reactants and desorption of intermediate products.

RGO‐supported Mo_2_C NPs were synthesized using a supercritical alcohol route, which was followed by carbothermal hydrogen reduction.^193^ This composite could be used as a catalyst for oleic acid deoxygenation to produce hydrocarbons with ≥85% yield and ≥90% hydrocarbon selectivity. This value is higher than those (yields = 18.5−50.3%) obtained for the Mo_2_C catalysts using other carbon substrates, including glassy spherical carbon, activated carbon, and mesoporous carbon. The high hydrocarbon yield for the Mo_2_C/rGO composite was attributed to the uniform distribution of Mo_2_C NPs on the 2D support as well as efficient transport of reactants imparted by the large pore size (≈9.7 nm) and slit‐like pore structure of the catalyst. The superior role of rGO as a catalyst support compared to other carbon materials (carbon nanotubes, activated carbon, and carbon black) has also been observed in the catalytic dehydrogenation of LiAlH_4_.^100^

Co_3_O_4_ NPs of 3.2–8.8 nm were attached on the surface of GO resulting from decomposition of cobalt nitrate in scCO_2_‐ethanol mixtures.^96^ This Co_3_O_4_/GO composite could catalyze the decomposition of ammonium perchlorate (AP) with lower decomposition temperature (297 °C) and enhanced exothermic heat (1591 J g^−1^) as compared to the values of 308 °C and 1448 J g^−1^, respectively, for pure Co_3_O_4_. Despite these preliminary results, mechanistic understanding of the catalytic decomposition of AP using Co_3_O_4_/GO is still unclear.

Local electronic structures of graphene and GO are easily modified by chemical doping with heteroatoms to yield metal‐free electrocatalysts. ScNH_3_ was reported to facilitate the formation of N‐doped graphene, which enables a 4e^−^ transfer oxygen reduction reaction (ORR) process in alkaline electrolyte. Such nonmetallic catalyst exhibited a more positive ORR potential (−0.3 V vs Ag/AgCl) and larger current density than the one prepared in NH_4_OH.^37^ This result may be explained by the relatively higher N content of the N‐doped G obtained in scNH_3_ than in NH_4_OH.

**Figure 9:**
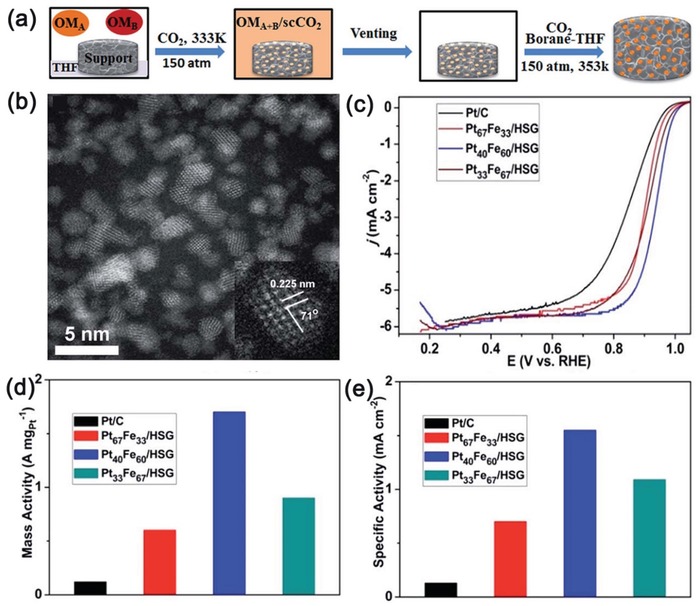
a) Schematic of multimetallic NPs/3D porous graphene (HSG) with SCF technique. b) HAADF‐STEM image of Pt_40_Fe_60_ NPs. Inset: Enlarged image of an individual NP with a plane and angle consistent with an *fcc* structure. c) ORR polarization curves of commercial Pt/C, Pt_67_Fe_33_/HSG, Pt_40_Fe_60_/HSG, and Pt_33_Fe_67_/HSG. d) Mass activities and e) specific activities measured at 0.9 V. Reproduced with permission.^107^ Copyright 2016, Royal Society of Chemistry.

In addition to abundance and environmentally friendly nature, graphene‐based materials possess outstanding electrical conductivity and high mechanical strength and stability, which are beneficial when used as catalyst supports in electrochemistry.^137^, ^196^, ^220^ A recent study by Lin et al. reported the synthesis of a series of Pt‐based/3D graphene composites by using an scCO_2_ technology (**Figure** **9**a,b).^107^ The as‐prepared Pt_40_Fe_60_/3D graphene delivered impressive ORR activities with 14.2‐fold enhancement in mass activity (1.70 A mg_Pt_^−1^ at 0.9 V vs RHE), 11.9 fold enhancement in specific activity (1.55 mA cm^−2^ at 0.9 V vs RHE), and higher durability compared with commercial Pt/C catalyst (Figure 9c–e). Such high performance was correlated with the use of the scCO_2_ approach which afforded highly dispersed and small metallic NPs in close contact with the 3D porous graphene support. Trimetallic PtFeCo/graphene cellular monolith (GCM) catalysts were derived using a similar SCF strategy. Improved ORR activities were obtained relative to bimetallic PtFe/GCM.^200^ Likewise, Pd NPs were uniformly deposited on the surface graphene in scCO_2_.^221^ The resulting Pd/G composite exhibited good electrocatalytic activity and stability for both formic acid and methanol oxidation reactions, surpassing the Pd/rGO, Pd/carbon nanotube, and Pd/XC‐72 hybrids.

SCF processing offers benefits for immobilization of semiconductors (such as TiO_2_,^202^ CdS,^222^ and ZnO^101^) on graphene (rGO or GO) with large contact surfaces for photocatalysis. For example, anatase TiO_2_ NPs of 5–10 nm were uniformly attached to the surface of graphene by solvothermal hydrolysis of titanium isopropoxide in supercritical ethanol.^202^ Such hybrid yielded nearly 100% degradation of methyl orange dye in 180 min, in contrast to that of 78% and 82% by pure TiO_2_ and P25. Fe‐doped TiO_2_ nanowires were grown on the surface of functionalized graphene sheets with —COOH groups in scCO_2_.^201^ The intimate contact between Fe‐doped TiO_2_ NPs and graphene benefited transfer of excited electrons from the Fe‐doped TiO_2_ anatase to graphene (with a work function around 4.2–4.5 eV). The band gap of Fe‐doped TiO_2_/graphene sheets (*E*_g_ = 2.25 eV at 0.6 wt% Fe doping) was reduced as compared to commercial TiO_2_ (*E*_g_ = 3.2 eV), which favors photocatalytic reactions in visible light. Interface synergistic effects between semiconductors and graphene (or rGO) likely contribute to separation of photogenerated carriers, thereby improving photocatalytic performance.^101^ The improved adsorption of dye molecules such as Rhodamine 123 dye and acetaldehyde by rGO (GO) also led to enhanced photocatalytic degradation of organic pollutants.^198^

ScCO_2_ was shown to enable phase conversion of 2H‐MoS_2_ into 1T‐MoS_2_ to form 2D 1T@2H MoS_2_ heterostructures.^181^ An electrode with such heterostructure displayed a photocurrent response of 1.2 × 10^−5^ A cm^−2^ for water splitting under visible light excitation, three times higher than that of 2H‐MoS_2_. Such enhancement was ascribed to the suppression of charge recombination due to the presence of metallic 1T‐MoS_2_. An even higher photocurrent response (10.6 × 10^−5^ A cm^−2^ in visible light) was achieved by using a 2D lateral WS_2_‐WO_3_·H_2_O/1T‐2H MoS_2_ heterostructure obtained in scCO_2_.^67^ The addition of graphene could further promote this phase transfer.^78^, ^184^ The resultant triphasic 1T@2H‐MoS_2_/graphene hybrid catalyzed H_2_ evolution reaction with H_2_ production rate reaching 19.82 mmol g^−1^ per hour under visible light irradiation in the presence of TEOA as a sacrificial donor.^78^ Alternatively, 2D lateral WS_2_/WO_3_·H_2_O heterostructures were fabricated with the assistance of scCO_2_. The potential of WS_2_ at the conduction band (CB) minimum (0.42 eV vs the normal hydrogen electrode [NHE]) is lower than that of WO_3_·H_2_O (1.26 eV), while the valence band (VB) maximum level of WO_3_·H_2_O (3.51 eV) is higher than that of WS_2_ (1.81 eV). Hence photogenerated electrons and holes accumulated at the CB of WO_3_·H_2_O and the VB of WS_2_, respectively. This response resulted in enhanced photocatalytic activity toward the degradation of methyl orange under irradiation with visible light.^35^

### 4.2 Batteries

Rechargeable batteries such as Li‐ion batteries (LIBs) and Na‐ion batteries (SIBs) store chemical energy with an ability to deliver electrical energy response by repeated charging–discharging processes. 2D nanosheets are showing promise in rechargeable batteries with better rate capability and higher cycling stability than bulk counterparts.

The use of graphene as either a cathode or an anode permits fast electron and ion transport in electrodes, enabling fast charge and discharge. As an anode material for LIBs, graphene exhibits a theoretical specific capacity of 744 mAh g^−1^, provided that lithium is bound on both sides of graphene to form Li_2_C_6_.^223^ RGO (initial discharge capacities of 540–1600 mAh g^−1^ at 50 mA g^−1^),^161^, ^224^ modified RGO,^74^, ^164^ and RGO‐based composite materials (such as graphene/Si,^203^ graphene/CoCO_3_,^206^ and graphene/LiFePO_4_ 32) may be promising alternatives for high‐performance LIBs. For example, hydrogen‐enriched rGO derived by reduction of double‐oxidized GO in scIPA exhibited a rather high reversible discharge capacity of 1331 mAh g^−1^ even after 100 cycles at a current rate of 50 mA g^−1^, which outperformed thermally reduced GO and scIPA reduced single‐oxidized GO electrodes.^164^ A large irreversible capacity decay was observed during the first cycle with an initial coulombic efficiency of 45.4%, as in the case of GO and other rGO samples, which was attributable to the formation of a solid electrolyte interphase (SEI) layer due to the presence of defects and reactions between oxygenated functional groups and lithium ions. The electrode showed both good rate and cycling performances with charge capacities of 193 and 167 mAh g^−1^ after 1000 cycles at 5 and 10 A g^−1^, respectively. The abundance of hydrogen‐terminated groups in rGO was suggested to be beneficial for the high Li‐ion‐storage capacity. Likewise, the hydrogen‐terminated groups and defects on the carbon sheets were also proposed to contribute to enhancement in Na ion uptake.^204^ A reversible capacity of 300 mAh g^−1^ at 50 mA g^−1^ and cycling stability up to 2000 cycles at 1–5 A g^−1^ were demonstrated for hydrogen‐enriched porous carbon nanosheets in Na ion batteries. Modification of graphene with 1‐pyrene sulfonic acid sodium salt (1‐PSA) in scH_2_O improved Li‐ion charge–discharge properties of exfoliated graphite by providing more active sites (sulfonate groups) for adsorbing lithium ions.^74^

One of the major issues when using metal and metal oxide NPs as electrode materials in LIBs is the significant volume change during charge/discharge, limiting cyclability. This issue can be alleviated by combination with conductive graphene materials with high surface areas.^225^ Indeed, Si nanowires–graphene composites prepared in supercritical hexane showed improved specific capacities (1400 mAh g^−1^ at 420 mA g^−1^) and cycling stability (up to 30 cycles) as compared to pure Si NWs (about 1030 mAh g^−1^ during the 30th cycle).^203^ This stability can be explained by the possibility that graphene in the hybrid accommodated the volume changes of Si nanowires during the lithiation/delithiation. Similarly, SnO_2_‐rGO composites synthesized in scMeOH also provided high cycling stability (531 mA h g^−1^ at 0.1 A g^−1^ after 1000 cycles), showing potential as an anode material in LIBs.^205^

**Figure 10:**
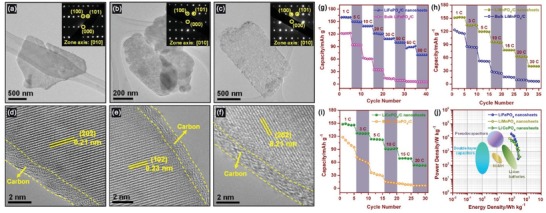
a–c) TEM images and d,f) HRTEM images of LiMnPO_4_/C (a,d), LiCoPO_4_/C (b,e), and LiNiPO_4_/C (c,f) nanosheets. Insets in panels (a–c): corresponding SAED patterns with a zone axis of [010]. Rate capabilities of g) LiFePO_4_/C, h) LiMnPO_4_/C, and i) LiCoPO_4_/C. j) A Ragone plot of LiMPO_4_/C nanosheets, in comparison with some advanced energy storage and conversion devices. Reproduced with permission.^60^ Copyright 2013, American Chemical Society.

Olivine‐type LiMPO_4_ (M = Fe, Mn, Co, Ni) nanosheets with exposed (010) surface facets were fabricated by employing a solvothermal lithiation process in SC ethanol–water solution (**Figure** **10**a–f).^60^, ^226^ These nanosheets exhibited better cycling stabilities as cathodes than their bulk materials in LIBs (Figure 10g–i). During the 50th cycle at 0.2 C, the LiFePO_4_/C, LiMnPO_4_/C, and LiCoPO_4_/C nanosheets (thickness: 3.7–4.6 nm) showed reversible discharge capacities of 163, 147, and 136 mAh g^−1^, corresponding to a capacity retention of 99.4%, 93.6%, and 88.3%, respectively.^60^ Perhaps more interestingly, the ultrathin nanosheet features facilitated fast lithium transport, affording high‐energy densities and excellent rate capabilities (e.g., 18 kW kg^−1^ and 90 Wh kg^−1^ at an 80 C rate for LiFePO_4_/C nanosheets) (Figure 10j), which are promising for energy storage and conversion devices with both high‐power and high‐energy densities. Li_2_MnSiO_4_ nanosheets of a few atom thick were also prepared in SC ethanol–water solution.^207^ This nanosheet structured cathode material showed two lithium extraction/insertion performances (a discharge capacity of ≈340 mAh g^−1^ at 45 ± 5 °C) with good cycle ability without any structural instability up to 20 cycles. V_2_O_5_ nanosheets that were prepared via an SCF route are another cathode alternative for LIBs.^63^ The nanosheets displayed a capacity of 90 mA h g^−1^ at 15 C and an almost 100% capacity retention after 200 cycles.

A recent report of preparing nanoparticulate SnO_2_ and depositing 1 nm SnO_2_ particles on graphene sheets exfoliated in scCO_2_ was developed for SIB anode applications.^227^ The reported synthesis was not entirely clear about whether nanoparticle synthesis was done before attempting to reach a supercritical state. The reaction purportedly contained 50 mL of ethanol, and this reaction was pressurized at 40, 60, and 80 °C in different preparations, and no mention of whether or not the critical conditions of their EtOH/CO_2_ mixture were exceeded was made.^227^ Critical parameters for ethanol/CO_2_ solutions have been determined.^228^ A mixture of 50 mL of ethanol in a 500 mL reactor pressurized to 10 MPa yields a mole fraction of CO_2_ of 0.86. The critical parameters of such a mixture of solvents are *T*_c_ = 77 °C and *P*_c_ = 12.6 MPa. These parameters were not attained in these experiments, so this processing should be termed “subcritical” or “near critical.” However, a better dispersity of nanoparticles obtained by pressurization was shown in TEM. Also, the specific capacity (mAh g^−1^) at “high” discharge rates up to 0 1 A g^−1^, was twice that measured for the “air” control as well as for CNT controls made with “ncCO_2_.”^227^ Therefore, these gas expanded “near critical, GA‐ncCO_2_‐ncEtOH, mixtures” were beneficial and should be so recognized.

### 4.3 Supercapacitors

Supercapacitors (or ultracapacitors), including electrical double layer capacitors and pseudocapacitors, are promising short‐term high power density storage devices for applications in between those requiring electrolytic capacitors and others requiring batteries. Graphene‐based materials obtained in SCFs have been exploited as supercapacitor electrode materials. Graphene aerogels prepared by scCO_2_ drying and H_2_ reduction provided a specific capacitance of 153 F g^−1^ and an energy density of 21.4 Wh kg^−1^ at a current density of 0.1 mA g^−1^ in an ionic liquid electrolyte (1‐ethyl‐3‐methylimidazolium bis[(trifluoromethyl)sulfonyl]imide) at 60 °C.^208^ The specific capacitance reached 90 F g^−1^ in an organic electrolyte (1 m MeEt_3_NBF_4_/PC). Graphene exfoliated in scNMP by a small scale homogenization process was used to make supercapacitors with a specific capacitance of 135 F g^−1^ in aqueous 6 m KOH.^154^ This capacitance dropped to about 100 F g^−1^ at current densities of 5 to 50 A g^−1^ with good cycling stability.

Nitrogen‐doped graphene sheets obtained in near‐critical fluids and SCFs^168^, ^169^, ^170^, ^171^ were used to obtain a maximum specific capacitance of about 290 F g^−1^ at 0.5 A g^−1^ in aqueous 1 m H_2_SO_4_.^170^ Capacitance retention of 98% with 100% coulombic efficiency was achieved over 1000 cycles of charge–discharge at 5 A g^−1^.^170^ It is not sure yet whether dimethylglyoxime‐saturated water is critical at only 400 °C, the maximal treatment temperature used with this oxime source. The functional groups and defects/edge sites were proposed to be more active in an acidic medium for pseudocapacitor reactions than in both alkaline and neutral media. The capacitive performance may be further improved by nitric acid treatment to generate pores.^169^ A fabricated symmetric supercapacitor cell using N‐doped graphene showed an energy density of 8 and 40 Wh kg^−1^ in aqueous 1 m H_2_SO_4_ and ionic liquid electrolyte, respectively. Note that supercritical reaction conditions and N‐precursors have a direct impact on the N‐content, N‐type doping, conductivity, and specific surface area, which profoundly affect capacitive behavior.

Incorporating metal oxides, such as MnO_2_,^98^ ZnO,^99^ and conducting polymers of polyaniline^211^ and polypyrrole^210^ into rGO with the assistance of scCO_2_ was shown to be capable of delivering high specific capacitances of 200 (based on cyclic voltammetry at 50 mV s^−1^ in 3 m KCl), 303 (based on galvanostatic charge–discharge at 10 A g^−1^ in 2 m KOH), 280 (at 1 A g^−1^ in 1 m H_2_SO_4_), and 144.6 F g^−1^ (based on cyclic voltammetry at 50 mV s^–1^ in 1 m H_2_SO_4_), respectively. High cycling stability was also claimed with a capacitance retention ratio of 98% for the ZnO/rGO composite electrode after 1000 cycles.^99^ This excellent performances of rGO hybrid electrodes can be attributed to a synergism between each single phase. RGO provides ample surface area and high electrical conductivity as well as superior mechanical and chemical stability. The oxide or polymer acts as a spacer to suppress graphene restacking and help create electrolyte‐accessible channels within the electrode. It is believed that SCF processing favors the formation of intimate contact between the oxide (or polymer) and graphene, which benefits enhancement of supercapacitor performance.^229^

ScCO_2_ has also been used to facilitate exfoliation of GO in aqueous dispersions for supercapacitor applications.^230^ The GO produced, in addition to being exfoliated, also acquired pores that provide active sites for charging and ion diffusion. Electrical double layer capacitances of 253 and 210 F g^−1^ were obtained at 1 and 16 A g^−1^, respectively.

### 4.4 Conductive Films and Electronic Devices

Direct exfoliation of graphite in SCFs obtained graphene films with high conductivity.^128^, ^133^ Current‐voltage (*I*–*V*) curves for vacuum‐dried graphene samples (at 200 °C) showed resistance in the range of 2–6 kΩ.^133^ An ohmic behavior was observed in a low voltage range, while nonlinear symmetric behavior and conduction increase in a high‐bias region were identified in the large voltage range. The current density was estimated to be over 1.0 × 10^9^ A cm^−2^. No annealing process is required using this SCF approach, which is quite useful for the fabrication of films on various substrates. The SCF method also showed advantages for the preparation of MoS_2_ thin films with remarkably better electrical transport performance (≈1530 cm^2^ V^−1^·s^−1^) than those obtained by many other techniques.^55^ A non‐linear *I*–*V* characteristic was reported for MoS_2_ devices. Such non‐linearity was assigned to various charge transport mechanisms such as space‐charge‐limited conduction, Fowler–Nordheim tunneling, and Poole–Frenkel conduction.

N‐doped graphene produced in scACN was demonstrated to display an *n*‐type behavior, in contrast to the *p*‐type field‐dependent behavior for pristine graphene.^138^ The sheet resistance of the N‐doped graphene was ≈300 Ω/⬜, indicating its good quality and high electrical conductivity.

### 4.5 Luminescence and Cellular Imaging

A diverse band structure among various 2D materials provides for many variations in optical properties in absorption and emission. Reviews of bulk and nanoscale luminescent materials,^231^ tuning luminescence in LDH materials,^232^ boron nitride nanomaterial luminescence,^233^ graphene‐based chemiluminescence sensors,^234^ layered rare‐earth hydroxides,^235^ and luminescence and associated mechanisms in graphene and related materials^236^ have been provided. Applications of nanosheets in cellular imaging have also been reviewed.^237^, ^238^, ^239^ Advantages that accompany SCF processing of 2D materials, mainly lack of chemical surface modification, are expected but have not yet been extensively realized.

More basic luminescence studies of nanosheets produced by SCF‐aided processing have begun but are not numerous. Yang and Hu doped CaTiO_3_ nanosheets with Eu^3+^ and found a maximum in emission intensity at a doping level of 0.2 mol%.^212^ These sheets were prepared from ethanol solution of Ca(NO_3_)_2_ solvo‐thermally with Ti(OC_4_H_9_)_4_ in scN_2_ at 1 MPa and 265 °C, and doping was done by including Eu(NO_3_)_3_ in the reaction. After venting, the nanosheets were collected and calcined in air for 4 h at 800 °C. A growth process that transformed nanoparticles into nanosheets was found that was driven by Ostwald ripening. Excitation of the resulting nanosheets at 466 nm produced a red emission at 616 nm.

Thangasamy and Sathish examined blue luminescence from nanosheets (nanoscrolls) of MoS_2_.^143^ This synthesis was done by sonicating powdered MoS_2_ in DMF, placing this reaction mixture in an autoclave at 400 °C for 30 min, and then quenching at 5 °C. The supernatant was collected and centrifuged to obtain the MoS_2_ nanosheets. The scrolling of nanosheets to form scrolls (and tubes) was explained as a process driven to minimize surface free energy. This minimization is a pervasive force in nature where Gibbs surface free energy is decreased by minimization of surface area to volume ratios and is a consequence of MoS_2_ units being constrained to a sheet structure after exfoliation. These authors noted a strong optical absorption band at 328 nm tailing into the visible as far as 500 nm. Their reported excitation spectrum indicated a peak in excitation at 360 nm and a blue emission peak at 420 nm. It was also mentioned that increasing the wavelength of excitation resulted in more extended wavelength emission, and some of these effects are dependent on particle size.^143^

Interestingly, Xu and co‐workers reported a novel scCO_2_ exfoliation process for MoS_2_ that produces stable nanosheets that are not scrolled.^79^ Their two‐phase process utilized MoS_2_ that was initially dispersed in aqueous ethanol, infused with scCO_2_ at 40 °C, and stirred to form Pickering emulsions of scCO_2_ and ethanol in an aqueous ethanol continuous phase, with scCO_2_ and ethanol solution droplets. These emulsion droplets are stabilized by MoS_2_ flakes that gradually exfoliate to stabilize more droplet interfacial area. The stability of the resulting MoS_2_ nanosheets, relative to the scrolls discussed above, can be rationalized by the much lower SCF temperature of this scCO_2_ process compared to the scDMF process. Xu and coworkers noted that such MoS_2_ nanosheets likely would be useful for cellular bioimaging applications, but did not report any.^79^ They did report luminescence properties of multicomponent emission spectra that shift red as excitation wavelengths shift from 300 to 550 nm. In their another work,^240^ polydisperse MoS_2_ nanosheets were prepared in scCO_2_/PVP/ethanol aqueous solution. Small nanosheets with lateral dimensions below 120 nm were used as fluorescent labels to perform lung cancer cell labeling.

Some demonstrations of using SCF‐processed nanosheets to label cells have been reported.^144^, ^241^ Anthracite coal was demonstrated by Poulin and co‐workers as a source of nanosheets using an scH_2_O process (374 °C, 22.1 MPa) that was mildly oxidizing. Under certain supercritical conditions, GO nanosheets were obtained as 3 nm diameter quantum dots in high yield (55%).^241^ These GO QDs were evaluated as labels in HeLa cells and found not to exhibit toxicity at up to 40 µg mL^−1^ and to provide a distinct blue fluorescence after 24‐h incubation with these cells. A separate scEtOH process (241 °C, 6.1 MPa) that was mildly reducing was found to produce graphenic ribbon‐like nanosheets at 6.8% yield. These ribbons produced electrically conducting films with a surface conductivity of about 0.1 mS/□. These different materials obtained with scH_2_O and scEtOH were obtained without using any strong acids or bases and yielded nanosheets in useful amounts.^241^

Sathish and co‐workers also reported an scDMF process (450 °C) for creating 5 nm diameter BN QDs from *h*‐BN.^144^ They reported a mechanism that proceeded through nanosheet formation by exfoliation followed by nanosheet disintegration into QDs. An excitation multiplet drove blue luminescence peaking at 414 over 270 to 380 nm. An exciting cell‐labeling phenomenon was found where the BN QDs stained Gram‐negative bacteria (*Escherichia coli* and *Pseudomonas aeruginosa*) but not Gram‐positive (*Staphylococcus aureus* and *Bacillus subtilis*). Epifluorescence microscopy could be used to differentiate Gram‐negative and Gram‐positive organisms in the same field because of this differential staining phenomenon. These authors hypothesized that this differential staining was due to an outer wall lipid layer not present in Gram‐positive bacteria.^144^

### 4.6 Sensors

2D materials have been extensively developed as wet chemical and gas phase sensors, and reviews of their applications focusing on GO,^242^, ^243^ rGO,^243^ and graphene,^243^, ^245^ chalcogenides,^244^, ^245^, ^246^, ^247^, ^248^, ^249^ oxides,^248^ and layer‐by‐layer^242^, ^250^ assemblies are available. In all electrochemical‐based sensing, it is important to maintain good electrical conductivity in a sensor‐intended composite material, similarly to 2D materials used in electrocatalysis.^2^

Only a few reports have appeared so far making use of SCFs in preparing such sensors from 2D materials. Wu and co‐workers prepared Pd nanoparticle (NP)–graphene composites using scCO_2_ to provide nanosheet separation and promote uniform distribution of suspended Pd NPs on these graphene nanosheets.^97^ These dispersed nanosheets are prevented from restacking tightly following CO_2_ removal by the adsorbed Pd NPs. Electrochemical sensing of ascorbic acid, dopamine, and uric acid was superior to graphene and “conventionally prepared” Pd/graphene electrodes using both cyclic voltammetry (CV) and differential pulse voltammetry.^97^ In this context, superior means higher sensitivity (1× to 4×).

Similar Pd–graphene composites prepared by scCO_2_‐assisted exfoliation and NP deposition were used to also examine effects of ionic liquids (ILs).^104^, ^213^ In a comparative study of graphene (G) and multiwalled carbon nanotubes (MWCNTs), Pd NPs were deposited with assistance from scCO_2_ (10 MPa, 50 °C). Electrodes were prepared as pastes by combining respective nanocarbon materials with isopropyl alcohol/Nafion solutions and depositing onto glassy carbon electrodes. IL‐containing examples were prepared with 1‐butyl‐3‐methylimidazolium hexafluorophosphate adsorbed into Pd/nanocarbon pastes. For ascorbic acid, dopamine, and uric acid, the electrodes without added IL, the Pd‐MWCNT electrodes were slightly to significantly more sensitive than the Pd‐G electrodes. However, the added ILs significantly increased sensitivities for both nanocarbons, relative to the IL‐free electrodes, and the G electrodes outperformed the MWCNT electrodes by 2× to 3×.^213^ In a related study, it was demonstrated that both glucose and ascorbic acid could be detected by such electrodes prepared with scCO_2_ processing and augmented with IL.^104^ Butylmethylpyrrolidinium‐bis(trifluoromethanesulfonyl)imide (BMP–TFSI) IL was found to be beneficial for detecting glucose and butylmethylpyrrolidinium‐dicyanamide (BMP‐DCA) IL worked well for detecting ascorbic acid. These were the first demonstrations of detecting these analytes in the presence of each other without using an enzyme‐based detection system.^104^ A glucose sensor based on Au nanoparticle–decorated graphene and activated with an IL is also an example of a useful heterostructure produced using scCO_2_ processing.^214^ This sensor was made at 50 °C and incubated at 10 MPa, and contained an MeOH/CO_2_ mixture at least 20% MeOH (*T*_c_ = 239 °C) by weight. Such a mixture is 74% mole fraction CO_2_ (relative to CO_2_ and MeOH) and has critical parameters of 71 °C and 13 MPa.^228^ Therefore, the results reported^214^ must be attributed to sub‐critical CO_2_. Ionic liquids have been found to be most beneficial in electrocatalysis as well.^251^

### 4.7 Flame Retardants

Fire resistance benefitting from incorporating nanosheet materials into polymers and composite materials has been recognized, and multiple reviews are available for graphene,^252^, ^253^, ^254^, ^255^, ^256^, ^257^ metal dichalcogenides,^258^, ^259^, ^260^ phosphates,^261^ layered double hydroxides,^262^ and phosphonates.^263^, ^264^ Only a few studies have utilized SCFs in purposely formulating fire retardancy in composite materials, but all composites incorporating inorganic nanosheets will have increased flame retardancy if their matrix or continuous phase is a combustible polymer. This generality can fail if the nanosheets, such as MnO_2_, discussed below, catalyze thermal decomposition.

Poly(styrene‐*co*‐acrylonitrile) nanocomposites with nanoclay were prepared by melt blending in an extruder to provide nanocomposite sheets. These sheets were then subjected to scCO_2_ and allowed to expand at sub‐critical 110 °C to produce foamed sheets. Nanoclay‐containing nanocomposites yielded decreased peaks of heat release rate, demonstrating efficacy in flame resistance relative to polymer‐only foam controls.^121^ More recently Hu and coworkers reported using scDMF to exfoliate graphite and MnO_2_ to produce graphene nanosheets and exfoliated MnO_2_ nanosheets.^265^ The scDMF also promoted redox changes and mixed valence Mn oxide formation in the form of mixed shape nanocrystals.^179^ These nanocrystals formed mixed suspensions with graphenic sheets to finally form heterostructured epoxy nanocomposites comprising heterojunctions of graphene–graphene and Mn oxide–graphene contacts. These same mixtures of nanofillers were subsequently used to formulate flame‐retardant epoxy nanocomposites.^266^ Relative to separate graphene‐epoxy and MnO_2_‐epoxy materials, mixed graphene/MnO_2_ epoxy resins exhibited a synergistic and increased degradation with increasing heating relative to separate nanofillers. However, a mixed graphene/MnO_2_ epoxy resin that also contained a phosphate amine component, DAP (diphenylamido phosphate), provided synergistically improved flame retardation. This combination incorporating phosphate resulted in increased “over char” retaining larger amounts of filler at up to 700 °C.^266^ The graphene‐only epoxy nanocomposite without DAP performed nearly as well as the mixed filler plus DAP system.

### 4.8 Lubricants

Graphite's long‐known efficacy as a lubricant has made examining nanosheets as lubricants a natural application for investigation. Batteas and co‐workers have reviewed using nanosheets of graphene, graphane, fluorographene, MoS_2_, WS_2_, *h*‐BN, and α‐Zr(PO_3_)_2_ to control interfacial friction.^267^ Updated mechanistic and wear analyses have recently been provided,^268^ and interactions of such nanosheets with various functionalization at fluid–fluid interfaces have been reviewed.^269^ NiCl_2_ was combined with citrate and surfactant (sodium dodecyl sulfate) in ethanol and then with GO and reducing agent (dimethyl borane, DMAB), and then this suspension was infused with scCO_2_ at 18 MPa and 100 °C. Controlled depressurization after 2 h yielded Sc‐Ni/GO nanosheets uniformly covered with nickel nanoparticles. These nanosheets were used as an additive to liquid paraffin (LP), lubricant, as were nickel nanoparticles, GO, and nickel nanoparticle–covered GO (Ni/GO) controls. The performance of the Sc‐Ni/GO as an additive resulted in a lower coefficient of friction (COF) and smaller wear scar diameters (WSDs)^270^ than any of the controls.^83^ Decreases in COF were 17% and 32%, respectively, relative to Ni/GO and LP controls, and decreases of 24% and 43% in WSD were reported, respectively, for these same controls. These improvements are highly significant for what is mostly a solvent effect.

A similar study by Su and co‐workers addressed lubrication by scCO_2_‐processed Ag nanoparticles dispersed on GO in 10W40 engine oil.^84^ Their tribology improvements for their Sc‐Ag/GO material, in 10W40, were not as significant as in the Sc‐Ni/GO‐LP case discussed above. A COF decrease of 7% relative to Ag/GO was not distinguishably greater than experimental uncertainties, but an improvement of 32% was seen relative to a 10W40 control. WSD decreases of 6% and 26%, respectively, were obtained relative to Ag/GO and 10W40 controls. These results, however, are more practically meaningful because 10W40 is a widely used engine oil. Another particularly useful aspect of using SCF‐generated nanosheets for lubrication and wear applications is that SCFs generally do not chemically modify nanosheet surfaces (in comparison to sonolysis chemistries that covalently attach solvent fragments to basal planes).^141^, ^142^

### 4.9 Patent Activity Overview

The Gulari and co‐workers patent documents discussed earlier^38^, ^48^ serve as a prominent reference for ensuing patenting activity for using SCFs to process 2D materials. The major focus has been on extending and refining the use of SCF to convert graphite and other graphene precursors to graphene powders and dispersions.^271^, ^272^, ^273^, ^274^, ^275^, ^276^, ^277^, ^278^, ^279^, ^280^, ^281^, ^282^, ^283^, ^284^, ^285^, ^286^

Several of these efforts disclosed using intercalation driven by agents other than SCF.^271^, ^285^, ^286^, ^288^ ScH_2_O was used as a reaction medium for controlling oxidation,^290^ and scCO_2_ was used as a medium in which to do surface functionalization with SF_6_, O_3_, and BX_3_.^294^ Their use in composites are also being explored,^288^, ^289^, ^290^ including aerogel formulation and drying.^291^, ^292^, ^293^ These studies and other processing patent disclosures are very briefly summarized in **Table** **5**.

**Table 5:** Summary of patenting activity for SCF‐processed 2D materials

Product 2D material	Source or starting materials	Role of SCF	Ref.
Graphene	Expanded graphite	Exfoliation agent	105, 271
Graphene	Graphite and “coating agent” (polymeric binder)	Intercalation and exfoliation	287
Graphene (oxidized)	Graphite	Intercalation and exfoliation	272, 273, 274, 275, 276, 277
Graphene	Graphite, solvent, surfactant	Intercalation and exfoliation	278
Graphene	Coke, coal	Intercalation and exfoliation	279, 280, 283, 284
Graphene	Reduced graphene oxide, alcohol	Exfoliation and drying	281
Graphene	Graphite	Intercalation and exfoliation	282
Graphene	Graphite, intercalant	Exfoliation	285, 286
Graphene‐polymer composite	Graphite, intercalant	Intercalation and exfoliation	288
Graphene‐VO aggregate	Graphene fluoride, graphene oxide	Intercalation and exfoliation	289
Graphene‐containing porous carbon	Graphite, water, H_2_O_2_, organic carbon precursors	ScH_2_O facilitates oxidation	290
Graphene and MoS_2_ containing aerogels	Graphite, graphene, MoS_2_, metal oxides, metal nanoparticles, carbon nanotubes	Supercritical drying	291
BN‐containing aerogels	BN, boron oxide, carbon	Supercritical drying	292, 293
Surface‐functionalized 2D materials	2D material, SF_6_, O_3_, BX_3_	ScCO_2_ used as reaction medium, intercalation, and exfoliation	294

## 5 Summary and Perspective of Future Developments

Experimental process development, introductory dynamical modeling, and several application reports show that SCF processing of 2D materials offers advantages and possibilities of overcoming some limitations that accompany subcritical shear processing. Greater exfoliation, so far, can be obtained by exfoliation in subcritical liquids, and applications such as RFID fabrication, thermal absorption, dispersion stability, and coloration likely cannot be improved by SCF processing. More highly value‐added applications that seek defect density minimization, larger sheets, and higher electrical conductivity likely will benefit from advanced SCF processing, but more process development is needed. It has become apparent that the existence of defects depends a great deal on the source and process by which a particular type of 2D material is prepared. This is particularly evident for graphene.

It appears that some synergistic effects can be realized by formulating SCFs with immiscible co‐solvents to make emulsification a process component. Certain other SCFs provide useful chemical effects with some 2D materials. The importance of chemical surface modification in promoting and stabilizing exfoliation cannot be overemphasized.

Some SCFs with mild (low) critical parameters provide exfoliation without any chemical effects, and for the case of scCO_2_, molecular dynamics simulations have provided some mechanistic understanding of scCO_2_ graphenic sheet interactions.^38^ Critical pressures must be sufficiently large to separate sheets by about a nanosheet thickness in order to develop a sufficient free energy barrier to reaggregation in the absence of additional stabilizers.

It is important to distinguish exfoliation processing of “nonreactive” SCFs from SCF processing that concomitantly uses chemomechanical activation such as ultrasonication and externally applied high shear (small media milling). X‐ray powder diffraction measurements should be taken at intermediate stages in between distinct processing regimens in order to better quantify intercalation and exfoliation events.

When sedimentation or centrifugation steps are included in a process, the amount of material removed should be quantified gravimetrically. This will allow more meaningful tracking of dispersion efficiency, and allow more meaningful yields to be tracked and reported. Obviously, processing that obviates any need for centrifugation separation of large fractions of 2D materials is preferred when possible and when economically accessible.

### 5.1 Promising Features

Supercritical fluids provide excellent mass transfer, gas‐like diffusion coefficients, and very low viscosities.^83^ Besides, they exhibit negligible surface tension, and this property ensures excellent wetting of all solid and liquid phases with which they contact. We are used to thinking in terms of thermal activation, and we have become skilled at analyzing Arrhenius behaviors to estimate activation energies. Activation volumes may also be significant and can be derived from supercritical high‐pressure measurements.^295^ It has been suggested that SCFs jam themselves into interlayer spaces to initiate exfoliation. A slightly more refined explanation is to consider activated separation of adjacent nanosheets along edges that is followed by adsorption of solvent molecules on nanosheet surfaces. When such adsorption or wetting occurs, it facilitates further exfoliation. Graphically, this process has been depicted for a case of polymer stabilized exfoliation,^296^ and it has been noted that adsorbed solvent acts as a temporal barrier to vdW re‐aggregation of separated nanosheets.^129^

A general discussion about using activation volumes for mechanistic studies has been provided by Wu and co‐workers.^297^ Such an approach is deemed particularly appropriate when various reaction properties vary significantly with pressure, and such a situation exists when SCFs are used to intercalate and exfoliate 2D materials or to surface modify such materials. We have discussed how simulation studies have shown that scCO_2_ stabilization of graphenic sheets depends on supercritical density,^129^, ^192^ and it seems that such a pressure‐dependent property is ideally suited for volume activation analysis.

The most crucial role for a solvent in multiphase suspensions and dispersions is not solubility per se but wetting. For many highly dispersed systems, lack of solubility is preferred because it blocks unwanted growth through Ostwald ripening. When such growth is desired, it is a simple matter to include co‐solvents that will promote solubility and ripening.

Solvation of nanosheet surfaces is another significant feature, and the vanishing of surface tension above a solvent's critical point makes spontaneous wetting of all available surfaces favorable. This wetting facilitates formation of physical mixtures of nanosheets and particles. Such mixtures also experience rapid interdiffusion of components because of low viscosity and high diffusion coefficients.

Using SCFs because they do not chemically transform nanosheets is motivating for their use, and SCFs that are unreactive indeed are good candidates. Some SCF processing of 2D materials, wherein critical parameters are “mild”, offer a promise of facilitating exfoliation without leaving surfactant, polymeric, or solvent residues that interfere and thwart essential transport properties such as electrical and thermal conductivities. In cases of chemically reactive SCFs that vary with SCF and nanosheet composition, specific chemistries can be induced. In these cases, chemical modifications of 2D nanosheets by some SCFs are intrinsic under critical conditions and promote ripening, redox chemistries, heterostructure formation and Ostwald ripening, and doping. Also, when sonolysis forces are present, untoward or advantageous surface modification can be induced.

Multiple studies of incorporating ultrasonication into high‐pressure SCF reactors have shown synergistic results. Coupling these two processing methods is very amenable to existing chemical processing technology. Coupling shear with SCF reactors has been demonstrated so far using two different shearing approaches, and for SCFs with “mild” critical parameters have performed satisfactorily.

### 5.2 Challenges

A continuing challenge in SCF applications to dispersing 2D nanosheets is to know analytically how much nanosheet material has been dispersed. It is more difficult to do simple UV–visible absorption measurements of supercritical^298^ nanosheet suspensions, than doing such measurements in a laboratory at ambient conditions, but cells suitable for such measurements are available. A particular limitation is to agree on what reference absorption coefficient is applicable for single sheet materials. A related challenge is to articulate what constitutes pristine single and few‐sheet layered materials. Wet chemical tests for particular types of surface functionality that can quantify effects based on Avogadro's number, *N*_o_, of species, or even (*N*_o_)^1/10^ will be helpful in characterizing defects per gram of material. Selected sampling by AFM or by HRTEM is much less statistically significant.

Mixed solvent systems are becoming more important, including pairs of immiscible solvents as well as pairs of miscible solvents. Even in cases of immiscible solvents, there is finite co‐solubility of each in the other, so care must be taken in learning how a second solvent affects a mixture's supercritical parameters. We have discussed two examples wherein significant synergisms cause mixtures of SCFs (CO_2_ and MeOH, CO_2_ and EtOH) to become subcritical at conditions thought to be critical. Process development should be done with solvent combinations where *T*_c_ and *P*_c_ have been determined as a function of mole fraction of each solvent component, so that criticality can be tuned with confidence.

All reactors operating at elevated temperature and pressure pose safety concerns, and these concerns are particularly justified when working with SCFs. An explosion of pressurized reactors and vessels can cause catastrophic injury.^299^, ^300^ Necessary safety awareness, training, and precautions should be used in such experimentation.^301^, ^302^, ^303^, ^304^

### 5.3 Future Developments

A coupling of shear forces using homogenization technology^305^, ^306^, ^307^, ^308^ with SCF processing has just been reported,^154^ and likely will become increasingly important. Homogenization is the most widespread emulsification technology used in industry and offers many design and implementation opportunities. Such processing involves impinging a multiphase fluid against a target or plate or through a rigid and porous material of high tortuosity, wherein a substantial pressure drop occurs before target impingement or across the porous material.

Advances in using co‐solvents that will serve as a useful continuous phase solvent post depressurization and is using suitable stabilizers useful in post depressurization will make particular dispersion applications more practical. These include applications wherein such stabilizers do not thwart any nanosheet–nanosheet interactions needed (such as electrical and thermal conductivity). Ultrasonication will be used to have more significant advantages in inducing designed surface modification^12^, ^14^, ^309^ by solvent radicals to facilitate stabilizing dispersions in desired co‐solvents.

Pressurization^114^, ^310^, ^311^ and depressurization processing steps will be coaxially coupled with particular subcritical liquids to produce uniform dispersions suitable for forming coatings and monolithic materials before thermally initiated free radical polymerization, UV photoinitiated polymerization, and condensation polymerization. Such processes will facilitate producing more advanced composite materials than are currently accessible by extruder‐based technologies.

## Conflict of Interest

The authors declare no conflict of interest.
